# Intensive early and sustained lowering of non–high-density lipoprotein cholesterol after myocardial infarction and prognosis: the SWEDEHEART registry

**DOI:** 10.1093/eurheartj/ehae576

**Published:** 2024-09-01

**Authors:** Jessica Schubert, Margrét Leosdottir, Bertil Lindahl, Johan Westerbergh, Håkan Melhus, Angelo Modica, Nilo Cater, Jonas Brinck, Kausik K Ray, Emil Hagström

**Affiliations:** Department of Medical Sciences, Cardiology, Uppsala University, 751 85 Uppsala, Sweden; Department of Cardiology, Skåne University Hospital, Malmö, Sweden; Department of Clinical Sciences, Faculty of Medicine, Lund University, Malmö, Sweden; Department of Medical Sciences, Cardiology, Uppsala University, 751 85 Uppsala, Sweden; Uppsala Clinical Research Center, Uppsala, Sweden; Uppsala Clinical Research Center, Uppsala, Sweden; Department of Medical Sciences, Uppsala University, Uppsala, Sweden; Sweden Medical Affairs, Pfizer AB, Stockholm, Sweden; US Medical Affairs, Pfizer Inc, New York, USA; Department of Medicine, Karolinska Institutet, Stockholm, Sweden; Department of Primary Care and Public Health, Imperial College London, London, UK; Department of Medical Sciences, Cardiology, Uppsala University, 751 85 Uppsala, Sweden; Uppsala Clinical Research Center, Uppsala, Sweden

**Keywords:** Myocardial infarction, Non–HDL-cholesterol, MACE, Death, Secondary prevention

## Abstract

**Background and Aims:**

Non–HDL-C provides an estimate of lipid-associated risk and is a secondary treatment target after myocardial infarction (MI). The aim was to study the relationship between non–HDL-C levels after MI and risk of adverse outcomes.

**Methods:**

From the SWEDEHEART registry, 56 262 patients with MI were included. Outcomes were major adverse cardiovascular event (MACE: death, MI, and ischaemic stroke), death, and non-fatal MI. Non–HDL-C was assessed at admission, 2 months, and 1 year. Target achievement (<2.2 mmol/L) of non–HDL-C, timing thereof, and outcomes were assessed.

**Results:**

During median follow-up of 5.4 years, 9549 had MACE, 5427 died, and 3946 had MI. Long-term hazard ratio (HR) for MACE in the lowest vs. the highest quartile of achieved non–HDL-C at 1 year was 0.76 [95% confidence interval (CI) 0.71–0.81]. Short-term results were consistent also when assessing non–HDL-C levels at 2 months, including early events up to 1 year (HR 0.80, 95% CI 0.68–0.92). Similar results were observed for all outcomes. Patients achieving both early and sustained targets had lowest risk of outcomes (HR 0.80, 95% CI 0.74–0.86) vs. patients achieving target early or late (HR for both 0.86, 95% CI 0.79–0.93).

**Conclusions:**

The lowest achieved levels both at 2 months and at 1 year of non–HDL-C were associated with better outcome. The lowest risk was observed when target was achieved within 2 months of MI and sustained thereafter. These findings challenge the current stepwise approach for cholesterol lowering after MI, which inevitably results in delaying goal attainment and possible harm.

## Structured Graphical Abstract

**Structured Graphical Abstract:**
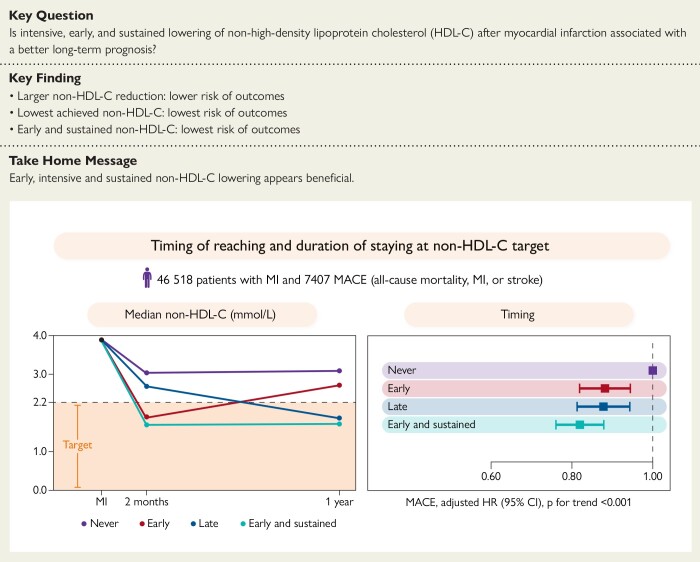
Non−HDL-C values at MI admission and early and late follow-up (left panel). Adjusted cox proportional hazard models for MACE for early, late, and early and sustained achievement of <2.2 mmol/L (right panel). Major adverse cardiovascular events are the composite outcome of all-cause mortality, myocardial infarction, or ischaemic stroke. CI, confidence interval; HR, hazard ratio; non−HDL-C, non−high-density lipoprotein cholesterol.

**See the editorial comment for this article ‘Non-HDL-cholesterol reduction: the challenges of applying clinical trial results in the real world’, by B. De Geest and S. Van Linthout, https://doi.org/10.1093/eurheartj/ehae577.**

## Introduction

Atherogenic lipoproteins include LDL, lipoprotein(a), and triglyceride-rich remnant particles, each of which carries a single apolipoprotein B (apoB) molecule. All apoB-containing lipoproteins are associated with an increased risk of atherosclerotic events.^1–4^ The overall atherogenic cholesterol content found in the apoB-containing lipoproteins can be estimated with the consolidated metric non–HDL-C. Most apoB-containing lipoproteins consist of LDL particles; hence, the majority of atherogenic cholesterol cargo is contained in LDL particles, and thus, most non–HDL-C comprises LDL-C. Consequently, the distribution of LDL-C is generally concordant with non–HDL-C. In scenarios in which the concentration of other apoB-containing lipoproteins is high, particularly when triglyceride-containing lipoproteins are abundant, LDL-C and non–HDL-C become discordant, and LDL-C inadequately captures the total lipid-associated risk, which is better approximated using either non–HDL-C or apoB. Of these, non–HDL-C is readily available and provided by subtraction of measured HDL-C from measured total cholesterol. Moreover, it can be reported in the non-fasting state and even when triglycerides are elevated.

Lipid-lowering therapies increase the expression or survival of the LDL receptor resulting in increased hepatic clearance of several apoB-containing lipid fractions, which results in lowering plasma levels of LDL-C, non–HDL-C, and apoB.^1^ Statins, for example, lower these by ∼50%, 46%, and 42%, respectively.^5–7^ This has largely led to the use of LDL-C as the principal target for lipid-modifying therapy, although equivalent non–HDL-C and apoB secondary targets are included in most evidence-based guidelines. With the global increase in obesity and diabetes, there are increasingly calls to use either non–HDL-C or apoB as a single, simple aggregate measurement of atherogenic risk. In the context of myocardial infarction (MI), initiation of high-intensity statins in-hospital results in greater reductions in all atherogenic lipids and reductions in cardiovascular events compared with moderate-intensity statins.^8^ Similarly, a combination of moderate-intensity statins plus ezetimibe was similarly superior to moderate-intensity statins alone.^9^ Furthermore, in survivors approximately ≥2 months after MI in whom LDL-C is not controlled, the addition of proprotein convertase subtilisin/kexin type 9 (PCSK9) inhibitors to statin therapy (with or without ezetimibe) reduces clinical events.^10^ These trials have been interpreted as recommending a stepwise approach starting with statins, with sequential addition of non-statin lipid-lowering therapies reserved for those who fail to achieve the requisite lipid target, potentially resulting in delays in lipid-target attainment and avoidable harm. We hypothesized that patients who achieved non–HDL-C targets (<2.2 mmol/L) early after MI and in whom the target was sustained would have the best outcomes vs. delayed target achievement (a proxy for the current stepwise approach).

## Methods

### Study population

This observational study used data from the Swedish Web-system for Enhancement and Development of Evidence-based care in Heart disease Evaluated According to Recommended Therapies (SWEDEHEART) registry. SWEDEHEART is a nationwide MI quality-of-care registry that records patient characteristics, medications, acute coronary care, and coronary interventions, as well as data on secondary prevention and outcomes.^11^ Data on all patients admitted with MI to any of the 74 coronary care units in Sweden are entered into the registry. This study included patients aged 18–79 years without previous atherosclerotic cardiovascular disease, admitted for a first MI between January 2005 and January 2022, and subsequently participating in the cardiac rehabilitation programme. Swedish cardiac rehabilitation programmes include a follow-up visit 2 months after discharge (timeframe 6–10 weeks) and after 1 year (timeframe 11–13 months) at which point data are entered into SWEDEHEART. Patients in this study had to have measurements of non–HDL-C at admission and at 1-year follow-up (see Supplementary data online, *Figure S1*). For the landmark analysis, patients had to have measurements of non–HDL-C at admission and at 2-month follow-up. If patients were hospitalized more than once during the observational period, only the first hospitalization was included. The registry was cross-referenced with three mandatory national registers held by the National Board of Health and Welfare (the patient register of all hospital admissions according to the International Classification of Diseases codes, the cause-of-death register, and the prescribed drug register containing data on all dispensed prescription drugs).

The Regional Ethics Committee in Stockholm approved the study in accordance with the Helsinki Declaration (approval numbers 2012/6013/2, 2018/1957-32, and 2019-04277).

### Exposures

Blood samples were drawn within 24 h of hospital admission for the index MI and in conjunction with the follow-up visits as per usual clinical care.

Non–HDL-C was calculated using the following formula:


[non–HDL−C]=[totalcholesterol]–[HDLcholesterol]

See Supplementary data for details on LDL-C calculation, statin treatment, and non–HDL-C threshold.

### Outcomes

Included outcomes were major adverse cardiovascular events (MACE; the composite of all-cause mortality, non-fatal MI, or non-fatal ischaemic stroke), all-cause mortality alone, and non-fatal MI alone. The study index date was the MI hospitalization date, and complete follow-up was until a fatal event occurred or April 2022, whichever occurred first. Assessments of outcomes were performed after the 1-year visit (see Supplementary data online, *Figure S2*). Outcomes were censored until the 2-month visit. Patients who had a non-fatal event between the 2-month and the 1-year visit were not excluded in the principal analysis. In a secondary analysis of short-term outcomes, the contribution of early non–HDL-C levels (as measured at the 2-month follow-up visit) on outcomes within the first year was assessed. The association between non–HDL-C levels at 2 months and all outcomes were assessed not restricting the population to survivors at 1 year. A landmark analysis was then performed assessing the contribution of changes in non–HDL-C at month 2 with early events occurring between 2 months and 1 year and late events after 1 year to internally validate our principal findings.

### Statistical analyses

Continuous variables are described by medians [interquartile range (IQR)]. Categorical variables are presented as counts and percentages. The difference in non–HDL-C between the MI hospitalization and the first and second follow-up visits was calculated. Kaplan–Meier survival probability estimates were calculated from the date of follow-up visit and to the total follow-up at 12 years for all outcomes, stratified by change in non–HDL-C. Censoring was done at the time of death, at the end of data capture, or after 12 years from the 1-year follow-up.

Cox proportional hazards regression models were used to assess the associations between the exposure and outcomes. A multivariable model, including all covariates simultaneously, was fitted. Study exposure and outcomes were assessed using Cox proportional hazards regression models presented as hazard ratios (HRs) and 95% confidence intervals (CIs). To allow for non-linear associations, continuous variables were entered as restricted cubic splines with five knots placed at the 5th, 27.5th, 50th, 72.5th, and 95th sample percentiles. Further, non–HDL-C was categorized in quartile groups and by <46% and ≥46% reductions from baseline. Hazard ratios were also calculated in linear models assessing 1 mmol/L reduction and in spline models comparing the 75th percentile with the 25th percentile. Owing to differential baseline characteristics, analyses were adjusted for the baseline value of non–HDL-C and demographic and clinical characteristics (see Supplementary data online, *Table S2*). The adjustment variables were chosen based on models derived from previous work on the same cohort.^8,12^ These are provided in detail in the Supplementary data. In sensitivity analyses, adjustment was not made for body mass index or history of diabetes as non–HDL-C may be considered a mediator between these variables and cardiovascular disease.^13^ Modelling was also performed adjusting results by year of inclusion, as treatment for acute coronary syndrome has evolved during the course of the study inclusion period.

Further, the HR for early (2-month visit) and late (1-year visit) reduction in non–HDL-C was calculated. Hazard ratios were also calculated in linear models for 1 mmol/L reduction in non–HDL-C in subgroups including age, sex, diabetes, body mass index, statin treatment at time of MI, and kidney function. The assumption of proportional hazards was assessed by visual inspection of Kaplan-Meier graphs and Schoenfeld residual plots (see Supplementary data online, *Figure S3*) for MACE at full follow-up. Missing covariate data were imputed with multiple imputations by chained equations with the assumption of data missing at random. In a sensitivity analysis, modelling was performed using a data set with complete covariate data. All analyses were performed at the Uppsala Clinical Research Center, Uppsala University, Uppsala, Sweden, using R version 4.2.3 (2023-03-15).

## Results

### Patient characteristics

A total of 56 262 patients had baseline and 1-year follow-up non–HDL-C measurements taken and were followed for a median of 5.4 years (IQR 2.6–8.9), yielding 328 390 patient-years of observation. Median age of the patients was 64 years (IQR 56–70) and 26% were women (*Table 1*). Median non–HDL-C at admission for index MI was 3.9 mmol/L (IQR 3.2–4.7), median LDL-C was 3.3 mmol/L (IQR 2.6–4.0), and 86% of the patients were statin-naïve (*Table 1*).

**Table 1:** Patient characteristics at admission

Variable	Overall (*n* = 56 262)	Quartile of non–HDL-C reduction between index myocardial infarction and 1 year			
		<0.7 mmol/L (*n* = 13 559)	≥0.7 to <1.5 mmol/L (*n* = 13 855)	≥1.5 to <2.2 mmol/L (*n* = 14 774)	≥2.2 mmol/L (*n* = 14 074)
**Admission characteristics**					
Age (years)	64 (56–70)	65 (58–70)	64 (57–70)	64 (56–70)	62 (55–68)
Female sex	14 488 (26)	4099 (30)	3387 (24)	3537 (24)	3465 (25)
BMI (kg/m^2^)	*n* = 52 961 27.1 (24.7–30.0)	26.9 (24.4–30.1)	26.9 (24.5–29.8)	27.1 (24.8–30.0)	27.4 (25.1–30.1)
**Medical history**					
Current smoker	*n* = 55 225 15 782 (29)	3961 (30)	4023 (30)	4021 (28)	3777 (27)
Hypertension	24 030 (43)	6672 (49)	5931 (43)	6026 (41)	5401 (38)
Diabetes mellitus	8629 (15)	3400 (25)	2155 (16)	1648 (11)	1426 (10)
History of heart failure	338 (1)	160 (1)	81 (1)	61 (<1)	36 (<1)
**Laboratory variables**					
Non–HDL-C (mmol/L)	3.9 (3.2–4.7)	2.9 (2.3–3.6)	3.4 (3.0–4.0)	4.1 (3.7–4.5)	5.1 (4.5–5.7)
LDL-C (mmol/L)	*n* = 55 840 3.3 (2.6–4.0)	2.4 (1.8–3.0)	2.9 (2.5–3.3)	3.4 (3.0–3.8)	4.3 (3.8–4.8)
eGFR (mL/min/1.73 m^2^)	*n* = 55 731 87 (74–96)	86 (71–95)	87 (74–96)	88 (75–96)	89 (76–97)
**Ongoing medication**					
Statin therapy					
None	48 333 (86)	8815 (65)	11 934 (86)	13 996 (95)	13 588 (97)
Low intensity	616 (1)	327 (2)	184 (1)	71 (1)	34 (<1)
Medium intensity	6166 (11)	3707 (27)	1473 (11)	606 (4)	380 (3)
High intensity	1147 (2)	710 (5)	264 (2)	101 (1)	72 (1)
Ezetimibe	310 (1)	166 (1)	60 (<1)	45 (<1)	39 (<1)
PCSK9 inhibitor	3 (<1)	2 (<1)	0 (0)	0 (0)	1 (<1)

Values are medians (interquartile ranges) and *n* (%) for categorical variables.

BMI, body mass index; eGFR, estimated glomerular filtration rate calculated by the Chronic Kidney Disease Epidemiology Collaboration equation; LDL-C, low-density lipoprotein cholesterol; non−HDL-C, non−high-density lipoprotein cholesterol; PCSK9, proprotein convertase subtilisin/kexin type 9.

At 1-year follow-up, median non–HDL-C level was 2.3 mmol/L (IQR 1.9–2.9), and LDL-C was 1.8 mmol/L (1.4–2.3). Median absolute decrease in non–HDL-C from index MI to 1-year follow-up was 1.5 mmol/L (IQR 0.7–2.2 mmol/L). Patients in the highest baseline non–HDL-C quartile had the largest absolute reduction in non–HDL-C at 1 year (2.7 mmol/L, IQR 2.1–3.3), compared with 0.5 mmol/L (IQR 0.1 mmol/L increase–1 mmol/L reduction) for patients in the lowest quartile.

Quartiles of non–HDL-C reduction of <0.7, ≥0.7 to <1.5, ≥1.5 to <2.2, and ≥2.2 mmol/L comprised 13 559, 13 855, 14 774, and 14 074 patients, respectively. Patients with the largest decrease in non–HDL-C at 1 year were more likely to be younger and had less prevalent diabetes and hypertension (*Table 1*).

Among patients with the largest non–HDL-C reduction at 1 year, 50% were treated with high-intensity statin as monotherapy, and 34% were treated with high-intensity statin in combination with ezetimibe at 1-year follow-up (see Supplementary data online, *Table S3*). The corresponding numbers for patients with the smallest reduction were 37% and 8%. Patients on high-intensity statin therapy (with or without ezetimibe) at both index MI and 1-year follow-up had a median non–HDL-C level of 2.2 mmol/L at 1 year, whereas patients on high-intensity statin at discharge but who had changed to a moderate-intensity statin at 1-year follow-up had a higher median non–HDL-C level of 2.8 mmol/L (see Supplementary data online, *Figure S4*). Patients on high-intensity statin therapy in combination with ezetimibe at index MI and at 1-year follow-up had a median non–HDL-C level of 2.1 at 1 year, while those on high-intensity statin but without ezetimibe had a median non–HDL-C level of 2.3 mmol/L at 1 year. Patients without statin therapy at discharge, 2 months, and 1 year after MI had a median non–HDL-C level of 3.0 mmol/L 1 year after index MI. Only 0.5% were on a PCSK9 inhibitor at 1-year follow-up. Proportions of patients on secondary prevention medications at discharge are shown in Supplementary data online, *Table S4*.

Temporal trends in pharmacotherapy, coronary interventions, and non–HDL-C levels are presented in Supplementary data online, *Table S5* and *Figures S5* and *S6*. Information on missing data is presented in Supplementary data online, *Figure S7*. Details on patients who died before 1-year follow-up are presented in Supplementary data online, *Table S6*.

### Outcomes according to non–HDL-C change

During follow-up, 9549 (17%) patients had a MACE, 5427 (10%) died, and 3946 (7%) had a non-fatal MI. After 1 year, MACE occurred at a rate of 3.07 (95% CI 3.01–3.13) per 100 patient-years of observation, all-cause mortality at 1.64 (95% CI 1.59–1.68), and non-fatal MI at 1.25 (95% CI 1.21–1.29).

#### Quartiles of non–HDL-C change

**Figure 1:**
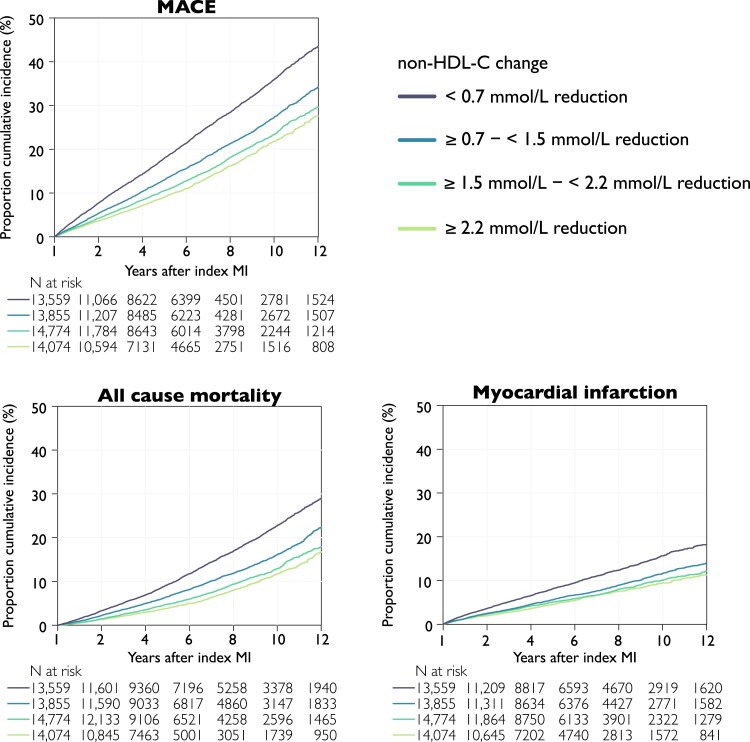
Cumulative incidence rates by outcome and change in non–HDL-C at 1 year after myocardial infarction. Kaplan–Meier curves of the cumulative incidence rates by quartile non–HDL-C reduction from index myocardial infarction to the follow-up visit. Log-rank test < .001 for all outcomes. Major adverse cardiovascular event is the composite outcome of all-cause mortality, myocardial infarction, or ischaemic stroke. MACE, major adverse cardiovascular event; non–HDL-C, non–high-density lipoprotein cholesterol

**Figure 2:**
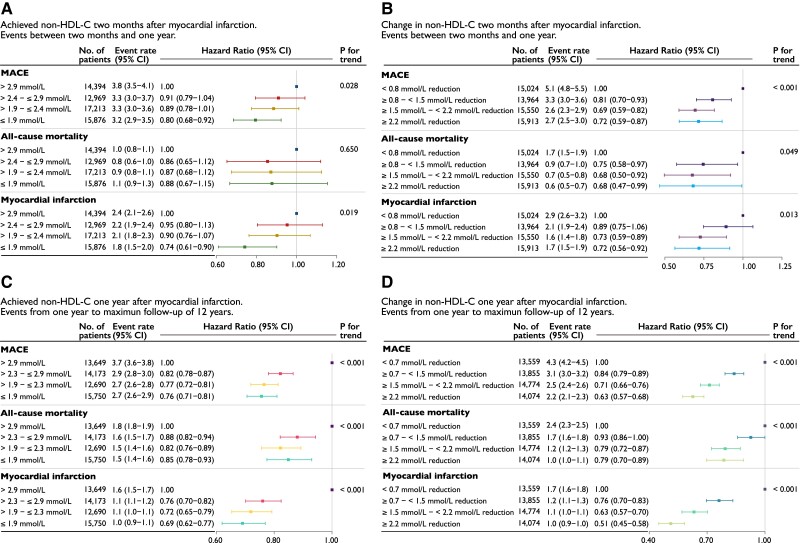
Event rates and hazard ratios by quartiles (*A* and *C*) of achieved non–HDL-C and (*B* and *D*) change in non–HDL-C between index myocardial infarction and 2-month follow-up (*upper* panels) and 1-year follow-up (*lower* panels). The *upper* panels shows events between 2-month follow-up and 1 year and the lower panels from 1 year to a maximum of 12 years. Event rate per 100 person-years. Models were adjusted for age at follow-up, statin intensity at admission, systolic blood pressure at follow-up, smoking at follow-up, sex, statin intensity at follow-up, body mass index at follow-up, history of diabetes, creatinine at admission, non–HDL-C at admission, and left ventricular ejection fraction at admission. Major adverse cardiovascular event is the composite outcome of all-cause mortality, myocardial infarction, or ischaemic stroke. CI, confidence interval; MACE, major adverse cardiovascular event; non–HDL-C, non–high-density lipoprotein cholesterol

The cumulative incidence rate of outcomes by quartile of non–HDL-C reduction from 1-year follow-up showed consistent curve separation with the largest reduction in non–HDL-C (≥2.2 mmol/L) associated with the lowest rates of events (*Figures 1 and 2D*). The incidence of MACE after 1 year decreased across quartile of non–HDL-C reduction, with rates of 4.3 (95% CI 4.2–4.5), 3.1 (95% CI 3.0–3.2), 2.5 (95% CI 2.4–2.6), and 2.2 (95% CI 2.1–2.3) per 100 patient-years, respectively, although the differences between quartiles 3 and 4 were less marked (*Figure 2D*).

The same trends were observed for all-cause mortality and non-fatal MI (*Figure 2D*). Relative to the quartile with the lowest non–HDL-C reduction, patients in the quartile of largest reduction had a 37% (HR 0.63, 95% CI 0.57–0.68) lower risk for MACE, 21% lower risk for all-cause mortality, and 49% lower risk for non-fatal MI (*P*_trend_ for all < .001; *Figure 2D*).

#### Per cent reduction ≥ 46%

Between the index MI and 1-year follow-up, 6631 (12%) patients had no reduction or an increase in non–HDL-C; 29 092 (52%) had a non–HDL-C reduction of <46%, and 20 539 (37%) had a reduction ≥46% (see Supplementary data online, *Figure S8*). Among the patients who achieved a reduction ≥46% at 1 year, use of high-intensity statins alone or high-intensity statins plus ezetimibe was higher, at 53% and 30%, respectively, vs. 46% and 12% among those who did not achieve a 46% reduction, and 31% and 6% among those who did not achieve any reduction. The magnitude of the percentage reduction in non–HDL-C was also inversely associated with the rates of MACE, all-cause mortality, and MI. As with absolute reductions, the difference in rates of events between those with a ≥ 46% vs. <46% lowering of non–HDL-C was less marked compared with a < 46% reduction vs. no change or an increase in non–HDL-C.

#### Achieved non–HDL-C at 1 year and clinical events

The quartiles of achieved non–HDL-C 1 year after MI were >2.9 (13 649 patients), > 2.3 to ≤2.9 (14 173 patients), > 1.9 to ≤2.3 (12 690 patients), and ≤1.9 mmol/L (15 750 patients).

**Figure 3:**
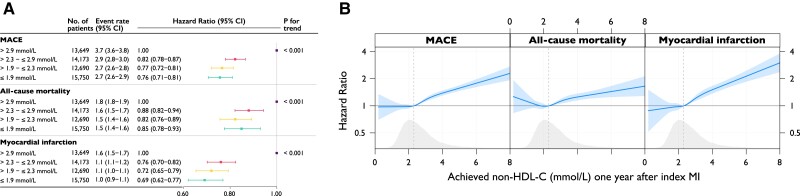
(*A*) Event rates and hazard ratios for quartiles of achieved non–HDL-C between index myocardial infarction and 1-year follow-up and (*B*) association between achieved values of non–HDL-C and outcomes. Event rate per 100 person-years. Models were adjusted for age at follow-up, statin intensity at admission, systolic blood pressure at 1-year follow-up, smoking at 1-year follow-up, sex, statin intensity at 1-year follow-up, body mass index at 1-year follow-up, history of diabetes, creatinine at admission, non–HDL-C at admission, and left ventricular ejection fraction at admission. MACE is the composite outcome of all-cause mortality, myocardial infarction, or ischaemic stroke. MACE, major adverse cardiovascular event; MI, myocardial infarction; non–HDL-C, non–high-density lipoprotein cholesterol

Across quartile of achieved non–HDL-C 1 year after MI, the risk of MACE and non-fatal MI was lowest among those in the lowest quartile, with a similar risk reduction in patients achieving a non–HDL-C below the median (*Figure 3A*).

Modelling the continuous relationship between achieved non–HDL-C at 1 year and outcomes in spline analysis, with the HR set to 1.00 at the median non–HDL-C level of 2.3 mmol/L, the risk of MACE increased monotonically with non–HDL-C level above the median. The hazards of all outcomes were linearly associated with the achieved non–HDL-C levels at 1 year after MI, with the lowest hazard being observed for patients achieving a non–HDL-C level of just below 2.0 mmol/L (*Figure 3B*).

#### Early and sustained achievement of non–HDL-C target

**Figure 4:**
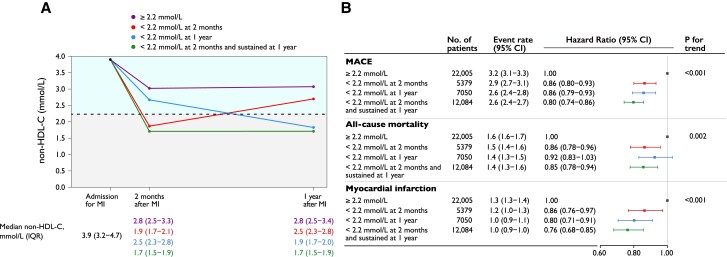
(*A*) Non–HDL-C values at myocardial infarction admission and early and late goal achievement. (*B*) Adjusted Cox proportional hazards models and event rates per 100 person-years are presented, adjusted for age at 1-year follow-up, statin intensity at admission, systolic blood pressure at 1-year follow-up, smoking at 1-year follow-up, sex, statin intensity at 1-year follow-up, body mass index at 1-year follow-up, history of diabetes, creatinine at admission, non–HDL-C at admission, and left ventricular ejection fraction at admission. MACE is the composite outcome of all-cause mortality, myocardial infarction, or ischaemic stroke. MACE, major adverse cardiovascular event; MI, myocardial infarction; non–HDL-C, non–high-density lipoprotein cholesterol; IQR, interquartile range; CI, confidence interval

In the subsample of patients (*n* = 46 518, 83% of the total population) with non–HDL-C assessed at baseline, 2-month, and 1-year follow-up, analysis of early and sustained target achievement was performed. In this population, 7407 patients had a MACE, 4115 died, and 3088 had a non-fatal MI. Failure to achieve a target non–HDL-C level of 2.2 mmol/L 1 year after index MI was compared with three scenarios: (i) achievement of target at 2-month follow-up that was not maintained at the 1-year visit; (ii) not achieving target at 2-month follow-up but achieving it at the 1-year visit; and (iii) achieving target at the 2-month follow-up that was sustained at the 1-year visit. The risk of MACE after 1-year follow-up was lowest for patients achieving the non–HDL-C target early and maintaining it at 1 year (HR 0.80, 95% CI 0.74–0.86), while the risk was similar if only achieving target early (HR 0.86, 95% CI 0.80–0.93) or late (HR 0.86, 95% CI 0.79–0.93; *Figure 4*, *Structured Graphical Abstract*).

#### Validation of contribution of early reductions to long-term benefits

A secondary analysis was performed in 60 452 patients who had a non–HDL-C assessment at baseline and at 2-month follow-up. Patients in the quartile with the largest reduction in non–HDL-C at 2 months had the lowest absolute and relative rates of all outcomes (see Supplementary data online, *Figure S9*). Consistent curve separation was observed for the quartile with the lowest reduction. Relative to the quartile with the smallest non–HDL-C reduction 2 months after MI, patients in the quartile with the largest reduction were at 34% (HR 0.66, 95% CI 0.61–0.71) lower risk for MACE (*P*_trend_ < .001; see Supplementary data online, *Figure S10*). The same trends were seen for all-cause mortality and non-fatal MI (see Supplementary data online, *Figure S10*).

The incidence rate of MACE and MI after 2 months decreased across achieved non–HDL-C quartiles, with the lowest rates observed among those in the lowest non–HDL-C stratum (see Supplementary data online, *Figure S11*). The risk for all-cause mortality was lowest among those in the lowest quartile with a similar risk reduction in patients achieving a non–HDL-C below the median (see Supplementary data online, *Figure S11*).

The relative impact of non–HDL-C levels at 2 months or change in non–HDL-C between index event and 2 months on *early* events (occurring between 2 months and 1 year) vs. *late* events (occurring after 1 year) was investigated in a landmark analysis censoring events before 2 months and after 1 year (*Figure 2*). The benefits of lower on-treatment non–HDL-C levels or larger changes in non–HDL-C on MACE, mortality, and MI were already apparent within the first year (10 months of exposure) with greater relative benefits observed after 1 year for each level of non–HDL-C exposure. This was qualitatively more apparent when comparing change in non–HDL-C rather than achieved levels (*Figure 2C* vs. *D*).

Hazard ratios for a 1 mmol/L reduction in non–HDL-C and comparing the 75th percentile of reduction to the 25th percentile are shown in Supplementary data online, *Figure S12*.

#### Sensitivity analyses

In a sensitivity analysis using complete data without imputation, the HRs at 1-year follow-up were near identical to the HRs using imputed data (see Supplementary data online, *Figure S13*).

To ensure consistent effects by non–HDL-C reduction, a series of interaction tests were performed using different patient characteristics and subgroups and outcomes at 1 year. Tests for interaction were performed for diabetes mellitus diagnosis, body mass index > 30 kg/m^2^, sex, statin use at admission, estimated glomerular filtration rate < 60 mL/min/1.72 m^2^, and age strata. No interaction was detected for risk of MI. There was an interaction for statin use at admission and MACE and all-cause mortality and for diabetes and all-cause mortality (see Supplementary data online, *Figure S14*).

The data were similar when omitting diabetes and body mass index from the adjustment model (see Supplementary data online, *Figure S15*). To assess whether time trends impacted outcomes, a sensitivity analysis was conducted adjusting for year of inclusion (see Supplementary data online, *Figure S15*). The results were near identical to the main analysis. Further, to assess whether the pattern of benefit from lower non–HDL-C was consistent over time, sensitivity analyses were performed stratifying the cohort into two discrete time periods. Event rates were, in general, lower in recent years, likely reflecting a higher proportion of patients on evidence-based pharmacological treatments as well as a higher proportion undergoing coronary revascularization (see Supplementary data online, *Table S5*). However, the lowest hazard was observed for early sustained goal achievement vs. never having achieved target (reference group) of HR 0.84 (95% CI 0.77–0.92) and 0.76 (95% CI 0.67–0.86), respectively (*P*_trend_ both <0.001; see Supplementary data online, *Figure S16*) irrespective of time period.

## Discussion

The European Society of Cardiology/European Atherosclerosis Society (ESC/EAS) guidelines^14,15^ recommend a stepwise approach to achieve lipid goals, starting with statins and adding non-statin lipid-lowering therapies. In patients at particularly high-risk, such as those with an MI, this stepwise approach might lead to delays in achieving lipid goals, especially as most patients admitted with MI are not receiving lipid-lowering therapy before the MI.^8^ The benefits or harms of early vs. delayed control of lipids after an MI are unknown. This observational study involving more than 55 000 patients with up to 12 years of follow-up in clinical practice after a first MI is the first analysis to investigate the potential impact of the timing and the magnitude of change in non–HDL-C levels after MI and subsequent risk of MACE, all-cause mortality, and non-fatal MI.

There were five key findings concerning the relationship between non–HDL-C at 1 year with the risk of adverse outcomes from 1 year onwards. First, the larger the decrease in non–HDL-C between the index MI and 1-year follow-up, whether assessed as a percentage change or absolute reduction, the greater the absolute and relative benefits with respect to subsequent risk of MACE, all-cause mortality, and MI. Second, lower achieved non–HDL-C levels were associated with lower risk of adverse outcomes, with the lowest risk observed in those achieving non–HDL-C just below ∼2 mmol/L. Third, achieving the ESC/EAS non–HDL-C (secondary) target (<2.2 mmol/L) early and sustaining it over the first year after MI was associated with the lowest risk of all outcomes compared with failure to maintain early reductions or delayed attainment of non–HDL-C targets. The latter finding suggests that achieving both intensive early and sustained lipid lowering after MI may be more beneficial than a stepwise approach, where target achievement would be expected to occur later. Fourth, strengthening this finding, in a landmark analysis looking at the relation between non–HDL-C levels at 2 months and events during first year after MI, the same associations were found. Furthermore, the same lowering of non–HDL-C provided greater relative benefits after 1 year. Fifth, the relationship between each 1 mmol/L lowering of non–HDL-C and lower risk of MACE, death, and MI was consistent across most subgroups, regardless of age, sex, and comorbidities.

From a lipid perspective, in the context of patients with a MI, if the ‘destination’ is to lower the risk of MACE, then the journey is via reductions in atherogenic lipids to attenuate, stabilize, and in some instances reverse the atherosclerosis process over time. The magnitude of the reductions in lipids and the achieved on-treatment lipid levels are determined by the potency and number of lipid-lowering medications used, with greater reductions achieved using more potent doses of statins and even greater reductions achieved when non-statins are added via combination therapy. In this regard, ezetimibe, bempedoic acid, and injectable therapies targeting PCSK9 are additive to the LDL-C lowering achieved with statins and, with the exception of inclisiran (currently in ongoing outcome trials), all have shown reductions in cardiovascular events. Data from statin trials, and more recently from trials of non-statins, show that the cardiovascular benefits when standardized per 1 mmol/L lowering of LDL-C are similar between therapies, with relative benefits proportional to absolute lowering (change/difference) in LDL-C.^1,16,17^ Furthermore, two head-to-head randomized controlled trials^18,19^ of high- vs. lower-intensity statins in conjunction with ezetimibe showed similar reductions in LDL-C and similar cardiovascular outcomes, indicating that the benefits are related more to the magnitude and duration of lipid reduction rather than how they are achieved.

Despite these data, implementation of evidence-based guidelines remains poor, with the DA VINCI registry (conducted between 2017 and 2018) and the SANTORINI registries (conducted between 2020 and 2021)^20,21^ suggesting that only about one in five patients achieve their LDL-C goals of <1.4 mmol/L (equivalent to a non–HDL-C of <2.2 mmol/L) on statin monotherapy even when on the most potent statin regimens. In both registries, goal attainment was higher when combination therapies were used. In this regard, our data show a low use of combination therapies and patterns of care largely reliant on statin-based monotherapy albeit at highest potency even 1 year after an MI. Patients achieving the largest percentage reductions in non–HDL-C tended to be on more potent lipid-lowering regimens including a greater use of combination therapy. Previous work in the SWEDEHEART registry on LDL-C changes after MI have shown that even after optimizing statin therapy, about 80% of patients do not reach LDL-C goals, and modelling shows that about another 20%–25% reach goals if ezetimibe is added, with the majority of the remainder predicted to achieve targets after the addition of PCSK9 monoclonal antibody.^22^ This is in keeping with similar simulation studies conducted in stable patients with coronary artery disease.^23^

An interesting observation in the present study is that although the cardiovascular benefits for quartiles 2–4 of change in non–HDL-C show a graded relationship with benefit vs. quartile 1 (smallest change), the difference between the top two quartiles of change (quartile 3 vs. 4) is less marked than between quartiles 1 and 2. This finding supports the notion that when reliant on statin monotherapy approaches alone, there is a ceiling of non–HDL-C reduction achievable and hence clinical benefit achievable at the level of the population, which could be overcome through use of combination therapies helping shift the population median and distribution of non–HDL-C towards lower levels. This is also in line with clinical trials showing improved cardiovascular outcomes with early addition of ezetimibe^9^ and with intensive vs. less-intensive statin therapies.^24–26^ This is further demonstrated in our study where patients on high-intensity statin and those on combination therapy had the largest reductions and the lowest event rates for all outcomes. The current treatment goal of LDL-C < 1.4 mmol/L in patients with atherosclerotic cardiovascular disease was established based on data from trials of add-on non-statin lipid-lowering therapies and average on-treatment levels achieved.^9,27,28^ A non–HDL-C level of 2.2 mmol/L is stated as the corresponding level to an LDL-C of 1.4 mmol/L in the same guidelines.^14^ Our study indicates that the relation between non–HDL-C and risk is monotonic to levels just below 2.0 mmol/L and is associated with the lowest risk of all outcomes, which is supported by data from PCSK9 inhibitor trials.^27^

Our analyses of the temporal relationship between change in non–HDL-C and outcomes suggest that early achievement of target non–HDL-C levels after MI, and its subsequent maintenance, was linked to better prognosis vs. achieving target levels at a later stage. Moreover, in landmark analyses, there appear to be meaningful, statistically significant, clinical benefits that increase over time, in support of the cumulative exposure hypothesis observed in clinical trials and by comparison with Mendelian randomization studies.^1,17^ Meta-analyses of randomized controlled trials further support early initiation of statin therapy and show a time-related impact of statin therapy on clinical outcomes of patients with acute coronary syndrome undergoing percutaneous coronary intervention.^29^ This is also consistent with the concept of variability or time-averaged change in lipids as key drivers of long-term risk of atherosclerotic cardiovascular disease, which some have termed ‘cholesterol years’.^1,17^ In a *post hoc* analysis of the Treating to New Targets (TNT) trial, a more uniform and less variable visit-to-visit LDL-C level was an independent predictor of cardiovascular events.^30^ The same was shown regarding non–HDL-C in an analysis of nine trials.^31^ While LDL-C is the primary treatment target, non–HDL-C changes are a reasonable alternative and extend our previous observations for LDL-C in the same registry.^8^ In fact, trials such as PROVE IT-TIMI 22 trial compared intensive with standard-dose statin therapy in patients with acute coronary syndrome within the previous 10 days and found a 1 mmol/L reduction in non–HDL-C and 1 mmol/L reduction in LDL-C resulted in similar reductions of risk for all outcomes.^32^ Similar findings were also observed in an early meta-analysis of 14 statin trials, in which a 1% decrease in non–HDL-C was associated with a 1% decrease in relative risk for coronary heart disease events.^33^ Further, in a meta-analysis of 49 trials with statin and non-statin lipid-lowering therapies, each 1 mmol/L reduction in non–HDL-C level was associated with 20% reduction in relative risk for major vascular events, similar to the relative risk reductions with LDL-C reduction of 1 mmol/L.^34^ This finding suggests that non–HDL-C is a reasonable alternative to LDL-C to assess response to lipid-lowering treatment and provide useful prognostic information, as has been performed in the present study.

These observations have practical implications for care pathways and implementation, where the stepwise statin-only first approach could be simplified by starting all statin-naive patients on a high-potency statin and ezetimibe pre-discharge, with early review at 2 months and the addition of further oral or injectable lipid-lowering therapies potentially shortening the time to achieve goals. For patients admitted with MI on lipid-lowering monotherapy (∼20% of the cohort) who tended to have more comorbidities and often did not receive a treatment change within 2 months (quartile 1 of non–HDL-C change) and had the worst outcomes, due consideration should be given to perhaps consider injectable therapies before discharge, which offer the largest additional reductions in lipids to statins as opposed to oral medications where the additional reduction would be half that of an injectable.^35^

In most individuals, around 90% of the circulating cholesterol burden is captured by estimating LDL-C levels. However, in certain cases, such as in patients with diabetes or metabolic syndrome, LDL-C is not fully representative of atherogenic burden since a large proportion of the cholesterol is found in the triglyceride-rich lipoproteins.^36,37^ This is important considering that >20%^38,39^ of the population with coronary heart disease events have diabetes and >75% are overweight or obese.^38,39^ Non–HDL-C may be considered a mediator between body mass index and history of diabetes and cardiovascular disease.^13^ Therefore, sensitivity analyses were made omitting them as covariates from the model, which yielded almost identical results. Non–HDL-C was shown to be a better predictor than LDL-C in patients receiving statin monotherapy in the TNT and IDEAL studies.^40^ This is consistent with findings from the ODYSSEY OUTCOMES trial, where patients with a recent MI who achieved the lowest levels of non–HDL-C on intensive dual or triple lipid-lowering therapy had improved prognosis compared with LDL-C when standardized by quantiles.^10^ Finally although non–HDL-C and apoB are closely correlated, often predicting outcomes to a similar extent,^40^ apoB may well be superior to other estimates including LDL-C and non–HDL-C.^41^ ApoB was, however, not routinely available, and hence, we were unable to perform studies of sufficient power that allowed comparison.

### Strengths and limitations

The present study was not aimed at comparing the prognostic utility of different atherogenic lipids but rather to maximize data and address the vexing issue of the relevance of timing, intensity, and persistence of lowering total atherogenic lipids and health outcomes after MI. Although the cholesterol content of lipid fractions such as lipoprotein(a), small dense LDL-C, and remnant particles are contributors to non–HDL-C, the cholesterol content of these particles underestimates their true risk associated with particle unit number or particle characteristics. In this study, no information was available on specific atherogenic particles or concentration of lipoproteins such as lipoprotein(a) or apolipoprotein and their individual prognostic utility could not be assessed.

A major strength of this study lies in its use of comprehensive quality-of-care registries that encompass all MI patients in Sweden. These registries not only provide a large and diverse patient population but also offer detailed phenotype information, allowing for a more nuanced analysis of the relationship between non–HDL-C levels and clinical outcomes. With around 75%–80% of all post-MI patients aged <80 years attending cardiac rehabilitation, paired with low numbers of missingness in the registry, the study allows for generalizability. The robust data linkage between registries, all with minimal missing data, enhances the reliability and completeness of the information, contributing to the overall validity of the findings. The prolonged observation period allows for a comprehensive exploration of the long-term implications of non–HDL-C levels on outcomes after MI.^42^ When exploring time trends, data show that the closer to the end of inclusion, the lower the achieved non–HDL-C level at follow-up, likely as a result of a higher prevalence of intensive lipid-lowering therapy (both high-intensity statin monotherapy and use of combination therapies). Further, higher prevalence of secondary prevention medication and revascularization after MI, as a sign of improved post-MI treatment, is illustrated by lower event rates in recent years. Nevertheless, when adjusting for year of inclusion, the results were identical, and the lowest HR was still observed for early sustained goal achievement when stratifying the cohort into two discrete time periods.

One notable limitation is the observational nature of the study, which inherently introduces potential biases and confounding factors. The main outcomes in this study were collected from the follow-up visits after index MI, thus all patients in the study needed to attend follow-up and have survived at least to 1 year after discharge. This introduces a selection bias and 1-year immortal time bias. To address these concerns, additional landmark and sensitivity analyses were conducted, and different adjustment strategies were employed, which provided consistent results, lending support to our observations. Further, a previous study from the same cohort showed that MI patients who are not followed in the SWEDEHEART cardiac rehabilitation programme due to old age or death before follow-up are more frail and have a higher non-cardiac mortality. Thus, the results of the study might not be applicable to these patients.^12^ While these efforts mitigate biases to some extent, the potential for residual confounding cannot be eliminated. However, the study’s alignment with data from both observational studies and clinical trials adds further credibility to its findings. Retrospective registry studies rely on existing data, which means that researchers have limited control over the variables included and the accuracy of the data collected. However, each of the registries has been highly validated and SWEDEHEART registry is undergoing monitoring in 2-year cycles.^11,43,44^ Treatment with PCSK9 inhibiting monoclonal antibodies was not considered, given the very low number of patients receiving this class during the study period. Data on therapy adherence was not available and the lipid-lowering analyses were treated as an ‘intention to treat’.

It is not possible to disentangle the reduction in non–HDL-C made by lifestyle changes from those achieved by pharmacotherapy in the current study. Lifestyle changes have other benefits beyond cholesterol lowering, and these changes will have an impact on outcome reductions beyond cholesterol lowering. However, lifestyle changes are for many hard to achieve and may take longer to achieve with respect to the magnitude of lipid lowering needed as compared with what can be achieved relatively quickly from pharmacotherapy by enhancing atherogenic lipid clearance via the LDL receptor. As the vast majority of non–HDL-C consists of LDL-C, and the magnitude of change in the latter dwarfs that achieved through diet and lifestyle, it follows that, the early reductions in non–HDL-C seen in this study are most likely due to pharmacotherapy.

Statins are associated with dose-dependent side effects that may occur more often early after initiating high-intensity treatment. Doubling the dose of statins yields on average a further 6% LDL-C lowering.^45^ If starting with combination therapy, for instance, high-intensity statins and ezetimibe, most patients are unlikely to experience adverse events and if they occur, using a combination lipid-lowering therapy allows for lowering statin doses but maintaining the effect of non-statin lipid-lowering therapy.

This study addresses the potential impact based on timing and achieved levels of non–HDL-C. We have not, however, assessed the safety, feasibility, and cost-effectiveness of earlier and more intense pharmacological lipid-lowering therapy. Further, our data are observational in nature, and randomized trial evidence addressing the question posed are lacking regarding the timing of reduction in lipids. It is however uncertain whether an ethics committee would agree to clinical equipoise and if patients would consent to a trial where in one group more intensive cholesterol-lowering treatment is withheld for one year. Hence, it is uncertain if such a trial would ever be acceptable or feasible.

## Conclusions

After a MI, the rates of cardiovascular outcomes and mortality were lowest among patients achieving the largest reductions in non–HDL-C and the lowest non–HDL-C levels down to ∼2 mmol/L. The lowest risk was observed when non–HDL-C targets or reductions were achieved early after MI and sustained thereafter, reflecting greater use of and maintenance of more potent lipid-lowering regimens. These data challenge the current paradigm of a statin-first stepwise approach towards lipid lowering after MI, which logically results in delayed control of lipids to one which implements earlier use of regimens capable of providing both early and if maintained sustained intensive lowering of non-HDL-C.

## Supplementary Material

ehae576_Supplementary_Data

## Declarations

### Data Availability

SWEDEHEART does not allow individual data sharing to a third party. Access to aggregated data might be granted following review by the SWEDEHEART steering committee.

### Pre-registered Clinical Trial Number

None supplied.
