# Supplementary material for: Intensive early and sustained lowering of non–high-density lipoprotein cholesterol after myocardial infarction and prognosis: the SWEDEHEART registry
Source: Eur Heart J. 2024 Sep 1;45(39):4204–15. doi: 10.1093/eurheartj/ehae576 (PMC11472424; doi:10.1093/eurheartj/ehae576)
Supplement: ehae576_Supplementary_Data [file ehae576_supplementary_data.pdf]

Supplementary data

Table of contents

LDL-C calculation....2

eGFR calculation 2

Definition of statin treatment 2

non-HDL-C threshold 2

Details on adjustment model covariates 3

Table S1. Statin intensity according to the American College of Cardiology/American Heart Association Guideline on the Management of Blood Cholesterol[1] 4

Table S2. Model covariates in statistical models 4

Table S3. Lipid-lowering intensity and non-HDL-C reduction between index myocardial infarction and 1 year 5

Table S4. Medications at discharge 6

Table S5. Proportion of secondary prevention at discharge by year of inclusion....6

Table S6. Patient characteristics at admission for index MI for patients who died within 1 year (*n*=7614) out of the total cohort....7

Figure S1. Study flowchart according to Consolidated Standards of Reporting Trials (CONSORT) 8

Figure S2. Study design....9

Figure S3. Assumption of proportional hazards for major adverse cardiovascular event at 12 years (Schoenfeld residual plots) 9

Figure S4. Changes in statin intensity and non-HDL-C from index MI....10

Figure S5. Median non-HDL-C levels per year of study inclusion....11

Figure S6. Proportion of lipid lowering medication by year of inclusion 12

Figure S7. Missing observations for covariates in adjustment model. 13

Figure S8. 46% reduction in non-HDL-C at 1 year 14

Figure S9. Cumulative incidence by quartile change in non-HDL-C between index MI and 2 months 15

Figure S10. Association between quartile change in non-HDL-C between index MI and 2 months and subsequent events....16

Figure S11. Achieved non-HDL-C at 2 months and subsequent events 17

Figure S12. Reduction in non-HDL-C and risk of outcomes....18

Figure S13. Reduction in non-HDL-C at 1-year, complete data 19

Figure S14. 1 mmol/L reduction in non-HDL-C between index MI and 1 year in subgroups 20

Figure S15. Association between achieved non-HDL-C levels at 1 year and outcomes in different adjustment models....22

Figure S16. Early and late goal achievement by inclusion year stratum....23

Disclosures of interest....24

Author contributions 25

Supplementary References 26

LDL-C calculation

LDL-C was estimated using the Martin−Hopkins equation[1]:

[total cholesterol] – [HDL-C] – [triglycerides/adjustable factor]

The adjustable factor is patient-specific for the ratio of directly measured triglycerides to very-low-density lipoprotein cholesterol. In a few cases, direct LDL-C analysis was used according to local standardized methods, which are subject to regular inspection and accreditation from a government authority.

eGFR calculation

eGFR, estimated glomerular filtration rate was calculated by the Chronic Kidney Disease Epidemiology Collaboration equation. eGFR < 60 mL/min/1.73m^2 was defined as reduced kidney function.

Definition of statin treatment

Information on lipid-lowering medication was retrieved from the prescribed drug register and statin intensity was calculated based on the prescribed dosage (*Table S1*). A patient was assumed to be taking lipid-lowering medication at admission if a drug dispensation was found within 6 months before their admission. A patient was considered treated with lipid-lowering medication at follow-up if a drug dispensation was found within 4 months before follow-up as well as 3 months after follow-up.[2] The intensity of the treatment was set according to the last dispense in the period carried forward, with a dispensation for lipid-lowering therapy usually lasting 90−100 days. Measures of persistence or adherence were not calculated.

non-HDL-C threshold

The risk of outcomes was analysed in relation to a <46% or ≥46% reduction in non-HDL-C from baseline. The threshold of a 46% reduction was chosen in analogy with the guideline-recommended decrease of 50% in LDL-C,[3] bearing in mind that the expected reduction in non-HDL-C with the same statin intensity is around 46%.[4–6]

Details on adjustment model covariates

The covariates entered into the models were chosen based on prior knowledge used in our previous studies on the same cohort [2,7] and from drawing a directed acyclic graph (DAG, see figure below). All measured confounders, i.e. covariates where data was available, were adjusted for. These covariates are age at 1-year follow-up, systolic blood pressure at 1-year follow-up, smoking at 1-year follow-up, sex, non-HDL-C at admission, left ventricular ejection fraction at admission, creatinine at admission, statin intensity at admission, statin intensity at 1-year follow-up, body mass index at 1-year follow-up, and history of diabetes (see *Table S2* below). Analyses were also made in unadjusted models. In sensitivity analyses, body mass index and diabetes were omitted from the main model since both variables influence lipid levels and, thus, non-HDL-C can be considered a mediator between these variables and cardiovascular disease.[8] Further, in an additional sensitivity analysis, year of inclusion was included in the model to take into account the advancement of acute MI care and secondary prevention during the observation period.

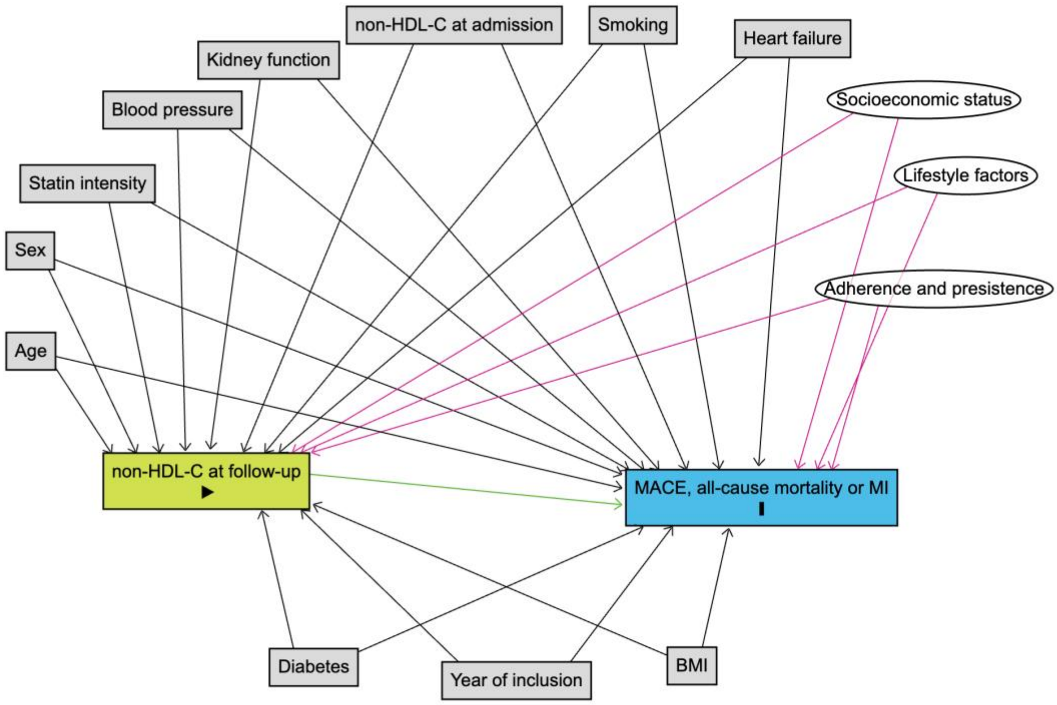

Directed acyclic graph (DAG) on confounders between levels of non-HDL-C at follow-up and adverse cardiovascular outcomes. Green box is exposure, blue box is outcome, grey boxes are measured confounders, white circles are unmeasured confounders. MACE is the composite outcome of all-cause mortality, myocardial infarction, or ischaemic stroke. MACE, major adverse cardiovascular event; MI, myocardial infarction; non-HDL-C, non-high-density lipoprotein cholesterol, BMI, body mass index.

Table S1. Statin intensity according to the American College of Cardiology/American Heart Association Guideline on the Management of Blood Cholesterol[1]

Statin	Statin intensity (mg)		
	Low	Moderate	High
Simvastatin	<20	≥ 20 to <80	≥80
Pravastatin	<40	≥ 40	–
Fluvastatin	≤40	>40	–
Atorvastatin	<10	≥10 to <40	≥40
Rosuvastatin	<5	≥5 to <20	≥20

Table S2. Model covariates in statistical models

Demographics	Medication	Laboratory variables	Comorbidities
Age at follow-up	Statin intensity at admission	Systolic blood pressure at follow-up	Smoking at follow-up
Sex	Statin intensity at follow-up	Creatinine at admission	History of diabetes
		Non-high-density lipid cholesterol at admission	Left ventricular ejection fraction at admission
			Body mass index at follow-up

Table S3. Lipid-lowering intensity and non-HDL-C reduction between index myocardial infarction and 1 year

	Quartile of non-HDL-C reduction (mmol/L)			
	**<0.7**	**≥0.7 to <1.5**	**≥1.5 to <2.2**	**≥2.2**
Number of patients	13 559	13 855	14 774	14 074
No lipid-lowering treatment	14%	3%	3%	3%
Monotherapy				
Low-intensity statin	1%	<1%	<1%	0%
Moderate-intensity statin	35%	33%	24%	13%
High-intensity statin	37%	48%	54%	50%
Ezetimibe	3%	1%	<1%	<1%
Combination therapy				
Low-intensity statin + ezetimibe	<1%	<1%	<1%	<1%
Moderate-intensity statin + ezetimibe	3%	2%	2%	3%
High-intensity statin + ezetimibe	8%	12%	17%	34%

Percentage of patients on different intensity of lipid-lowering treatment 1 year after myocardial infarction according to non-HDL-C reduction during the same period. Non-HDL-C, non-high-density lipoprotein cholesterol.

Table S4. Medications at discharge

Variable	Overall (n = 56 262)	Quartile of non-HDL-C reduction between index myocardial infarction and 1 year			
		<0.7 mmol/L (n = 13 559)	≥0.7 to <1.5 mmol/L (n = 13 855)	≥1.5 to <2.2 mmol/L (n = 14 774)	≥2.2 mmol/L (n = 14 074)
Blood pressure agents					
ACE-inhibitor	35,703 (64)	8240 (61)	8841 (64)	9605 (65)	9017 (64)
Angiotensin II receptor blocker	10,804 (19)	2994 (22)	2591 (19)	2665 (18)	2554 (18)
Beta blocker	48,797 (87)	11,898 (88)	12,180 (88)	12,773 (87)	11,946 (85)
Calcium blocker	6955 (12)	2080 (15)	1669 (12)	1680 (11)	1526 (11)
Antiplatelet agents					
Aspirin	54,289 (97)	12,870 (95)	13,407 (97)	14,309 (97)	13,703 (97)
Clopidogrel	18,753 (33)	5898 (44)	5159 (37)	4517 (31)	3179 (23)
Ticagrelor	31,812 (57)	5927 (44)	7386 (53)	8919 (60)	9580 (68)
Antidiabetic treatment					
Oral antidiabetic	5188 (10)	2053 (16)	1250 (10)	987 (7)	898 (7)
Insulin	3297 (6)	1500 (11)	804 (6)	543 (4)	450 (3)

Values are medians (interquartile ranges) and n (%) for categorical variables.

Table S5. Proportion of secondary prevention at discharge by year of inclusion

Year of inclusion	2005	2006	2007	2008	2009	2010	2011	2012	2013	2014	2015	2016	2017	2018	2019	2020	2021
Coronary intervention																	
PCI	69%	70%	70%	71%	72%	74%	77%	77%	79%	81%	81%	80%	80%	79%	81%	80%	78%
CABG	7%	8%	8%	7%	7%	5%	6%	7%	6%	6%	6%	7%	7%	7%	8%	7%	9%
Antiplatelet therapy																	
Clopidiogrel	85%	87%	87%	89%	89%	87%	83%	44%	20%	14%	11%	8%	8%	9%	9%	9%	11%
Ticagrelor	0%	0%	0%	0%	0%	0%	1%	44%	71%	77%	81%	84%	83%	83%	82%	82%	78%
Aspirin	96%	97%	97%	98%	97%	98%	98%	97%	97%	97%	97%	97%	96%	96%	96%	94%	94%
Other evidence based pharmacotherapies																	
Betablocker	92%	91%	92%	93%	93%	93%	92%	92%	91%	90%	90%	89%	88%	79%	76%	74%	74%
ACE I or AII blocker	67%	65%	74%	74%	78%	82%	82%	84%	84%	84%	85%	85%	85%	85%	84%	86%	86%

PCI, percutaneous coronary intervention; CABG, coronary artery bypass grafting; ACE I, Angiotensin-converting enzyme inhibitor; AII blocker, angiotensin II receptor blocker.

Table S6. Patient characteristics at admission for index MI for patients who died within 1 year (*n*=7614) out of the total cohort

Admission characteristics	
Age (years)	80 (72–85)
Female sex	3236 (43%)
Body mass index (kg/m^2)	*n*=9191<br>25.1 (22.5–28.3)
Medical history	
Current smoker	*n*=8441<br>1367 (20%)
Hypertension	2148 (28%)
Diabetes mellitus	1152 (15%)
History of heart failure	350 (5%)
Laboratory variables	
Non-HDL-C (mmol/L)	3.2 (2.5–4.0)
LDL-C (mmol/L)	*n*=7705<br>2.6 (2.0–3.4)
eGFR (mL/min/1.73^2)	*n*=7685<br>61 (43-81)
Ongoing medication	
Statin	
None	6348 (83%)
Low intensity	181 (2%)
Medium intensity	955 (13%)
High intensity	130 (2%)
Ezetimibe	33 (<1%)
PCSK9 inhibitors	0 (0%)

Values are median (interquartile range) or n (%) for categorical variables. eGFR, estimated glomerular filtration rate calculated by the Chronic Kidney Disease Epidemiology Collaboration equation; LDL-C, low-density lipoprotein cholesterol; MI, myocardial infarction; non-HDL-C, non-high-density lipoprotein cholesterol; PCSK9, proprotein convertase subtilisin/kexin type 9.

Figure S1. Study flowchart according to Consolidated Standards of Reporting Trials (CONSORT)

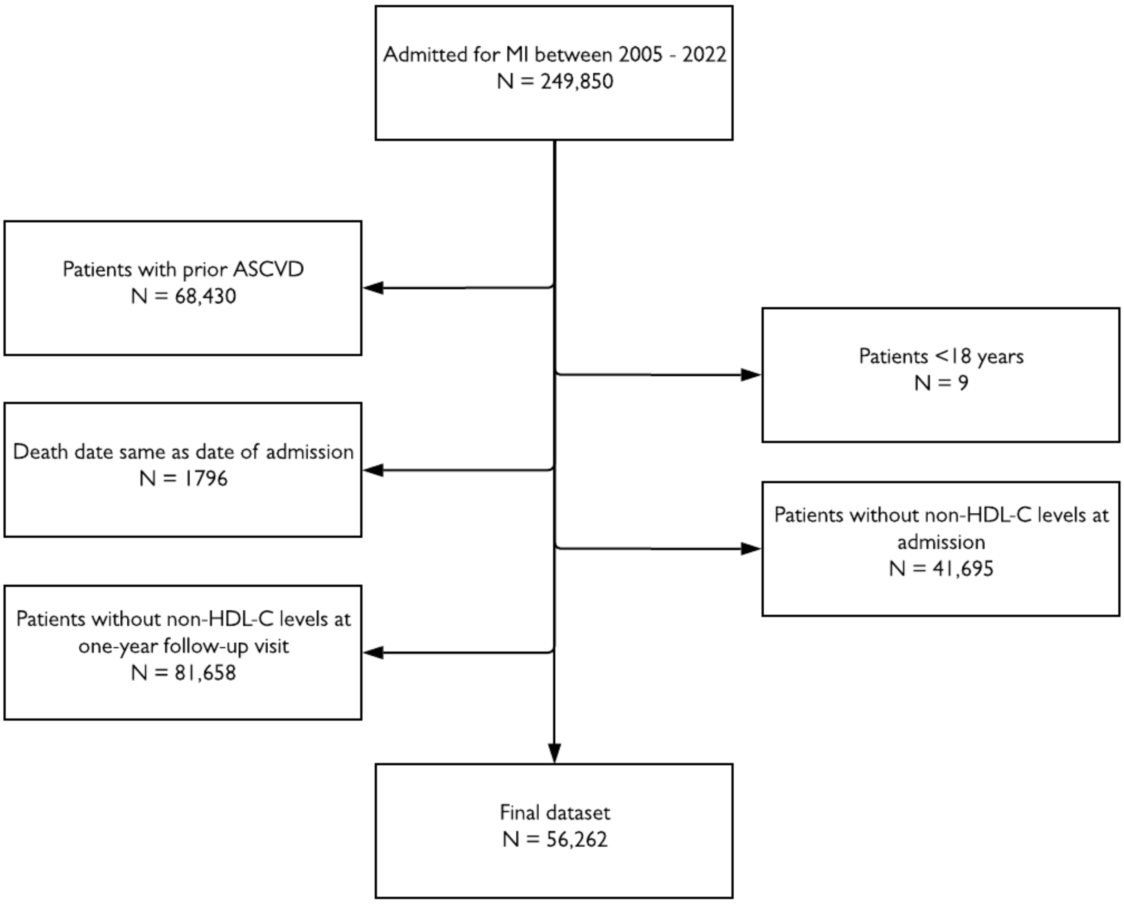

ASCVD, atherosclerotic cardiovascular disease; MI, myocardial infarction; non-HDL-C, non-high-density lipoprotein cholesterol.

Figure S2. Study design

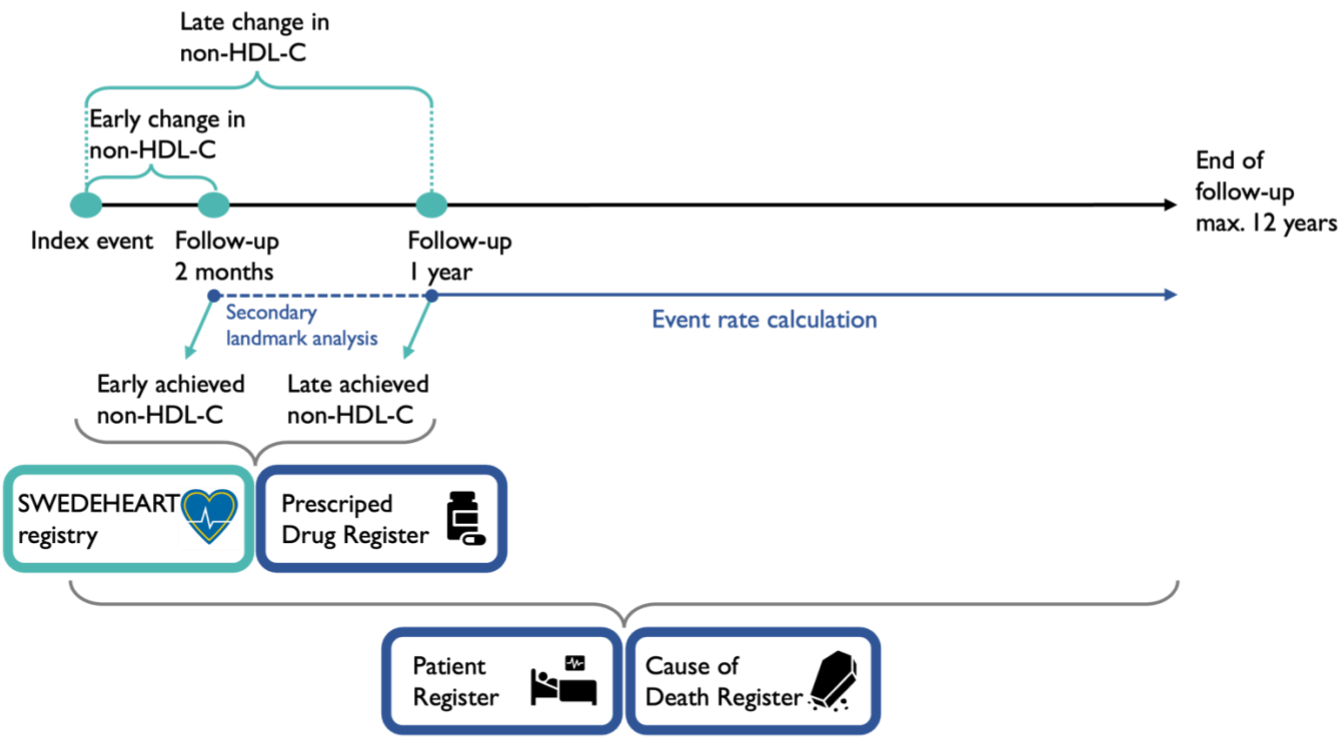

Non-HDL-C, non-high-density lipoprotein cholesterol.

Figure S3. Assumption of proportional hazards for major adverse cardiovascular event at 12 years (Schoenfeld residual plots)

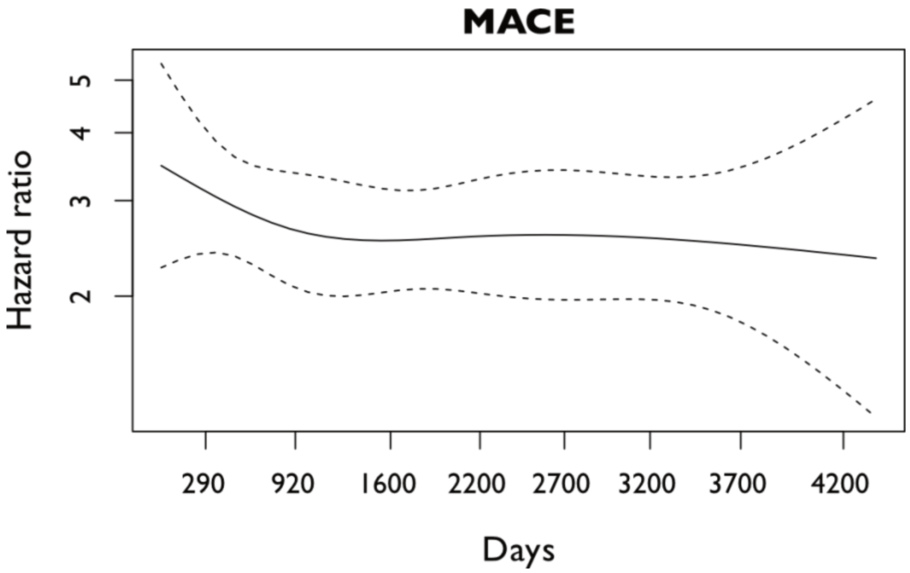

Axes are on a logarithmic scale.

Figure S4. Changes in statin intensity and non-HDL-C from index MI

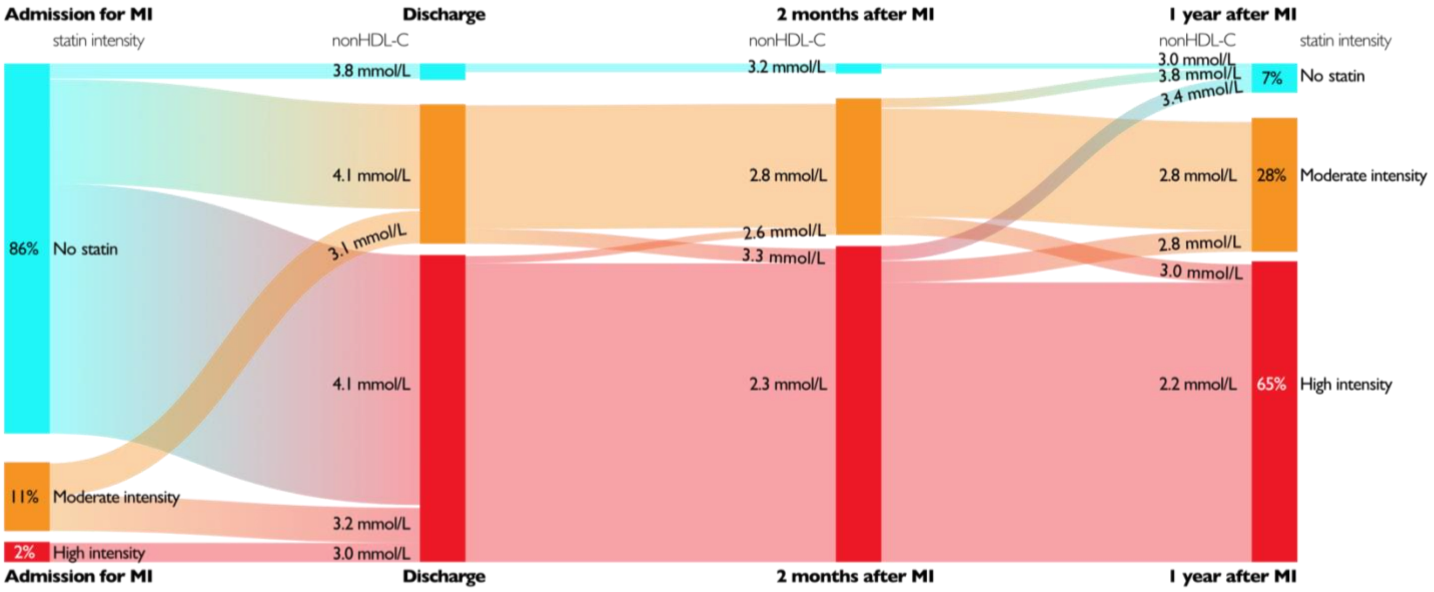

Statin intensity at admission, discharge, 2 months, and 1 year after index MI, and their corresponding non-HDL-C levels. The low-intensity group was removed due to few numbers. MI, myocardial infarction; non-HDL-C, non-high-density lipoprotein cholesterol.

Figure S5. Median non-HDL-C levels per year of study inclusion

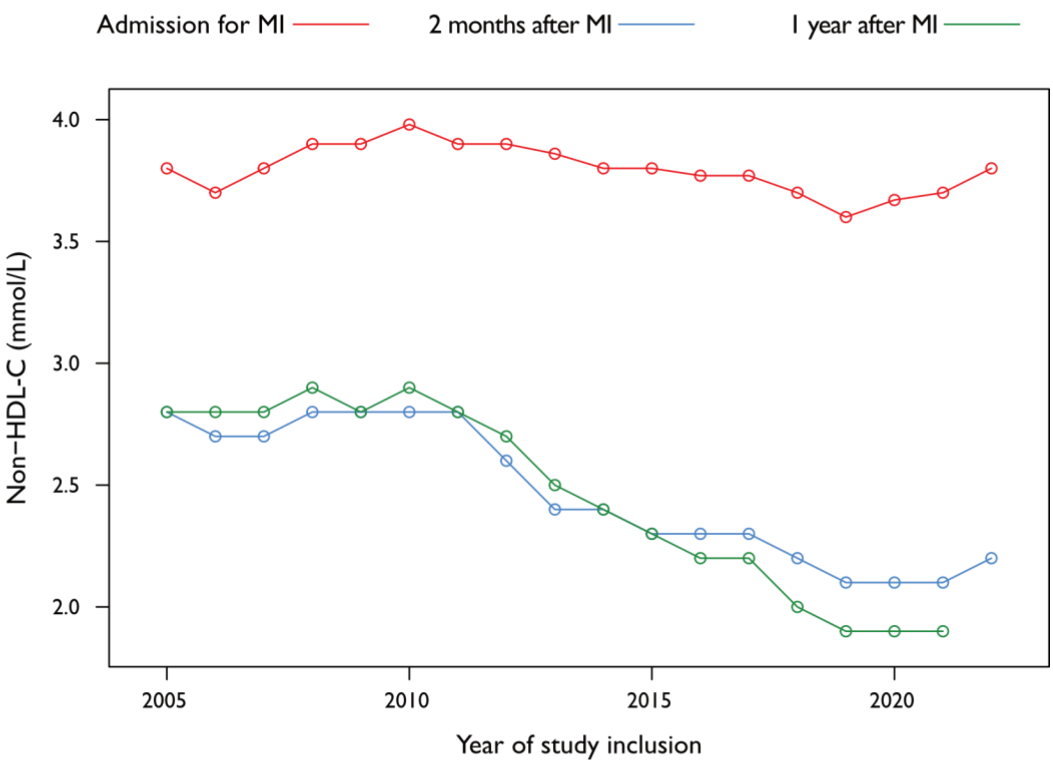

Median levels of non-HDL-C at admission (red), 2-months follow-up (blue) and 1-year follow-up (green), per year of study inclusion.

Figure S6. Proportion of lipid lowering medication by year of inclusion

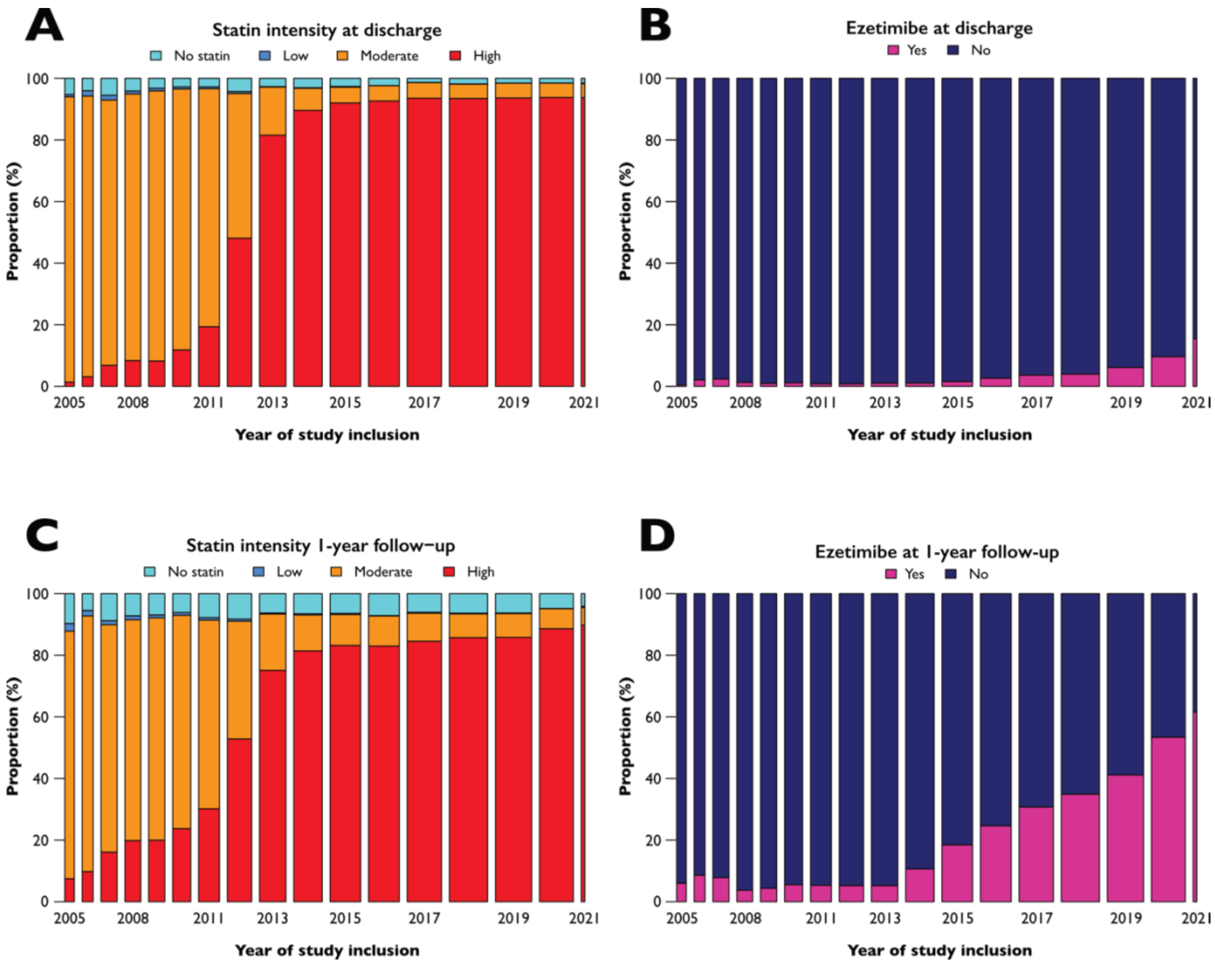

Proportion of patients on various intensity of statin therapy (A) and ezetimibe (B) at discharge and at 1-year follow-up (C, D) by years of inclusion. Width of bars represents the proportion of patients in the study.

Figure S7. Missing observations for covariates in adjustment model.

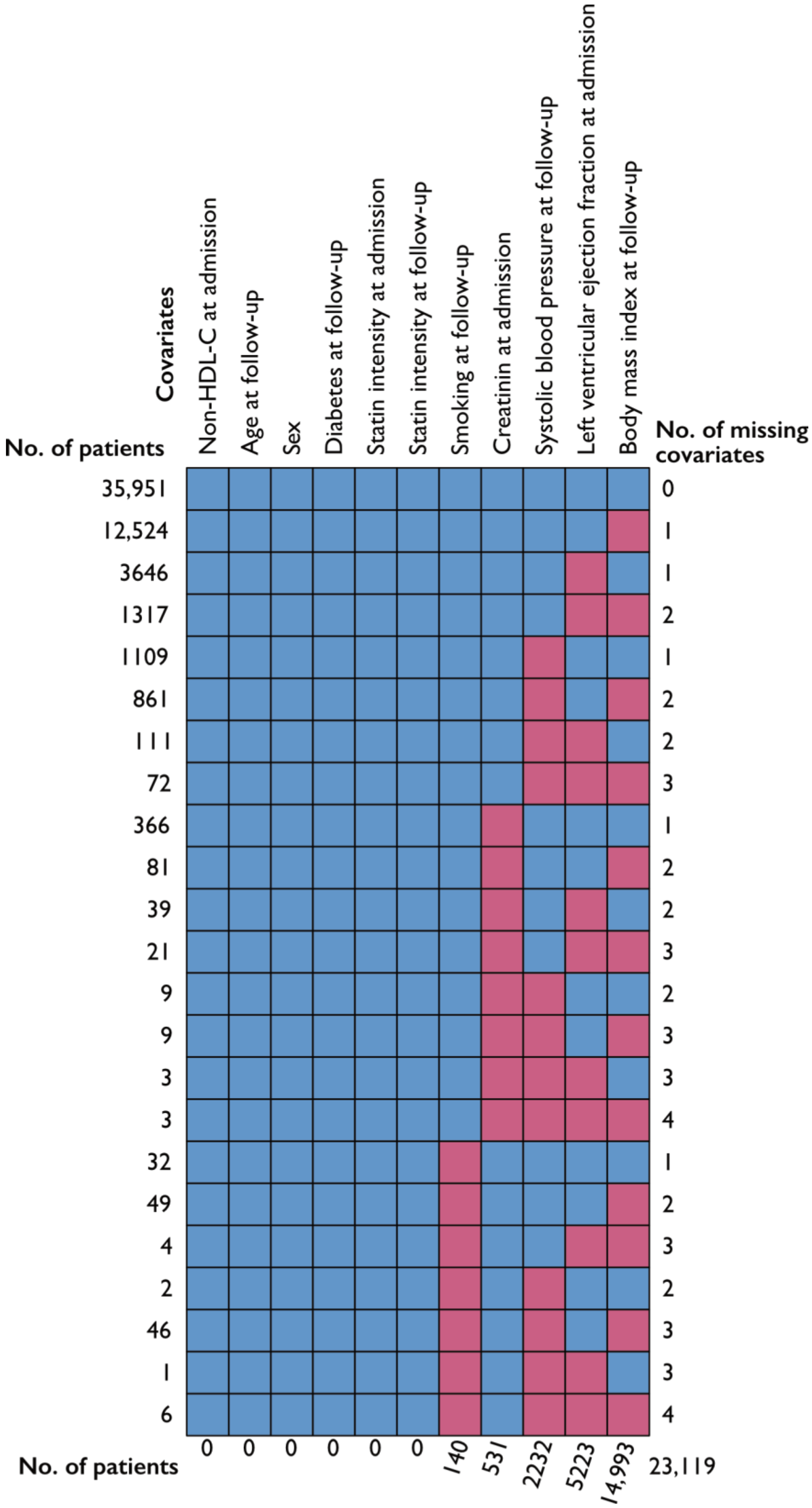

Figure S8. 46% reduction in non-HDL-C at 1 year

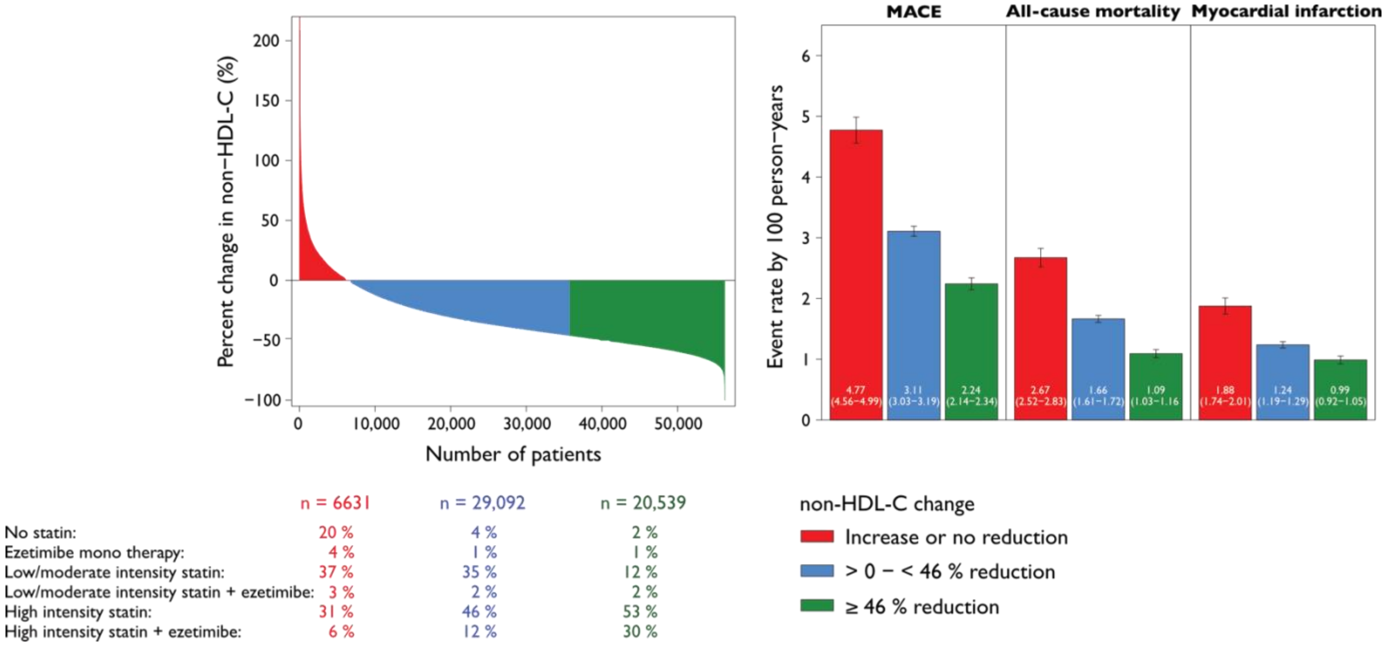

Waterfall plot for change in non-HDL-C reduction and event rates with 95% confidence intervals. Data are stratified by increase or no reduction (red), >0% and <46% reduction (blue), and ≥46% reduction in non-HDL-C (green) between index MI and 1 year. MACE is the composite outcome of all-cause mortality, myocardial infarction, or ischaemic stroke. MACE, major adverse cardiovascular event; MI, myocardial infarction; non-HDL-C, non-high-density lipoprotein cholesterol.

Figure S9. Cumulative incidence by quartile change in non-HDL-C between index MI and 2 months

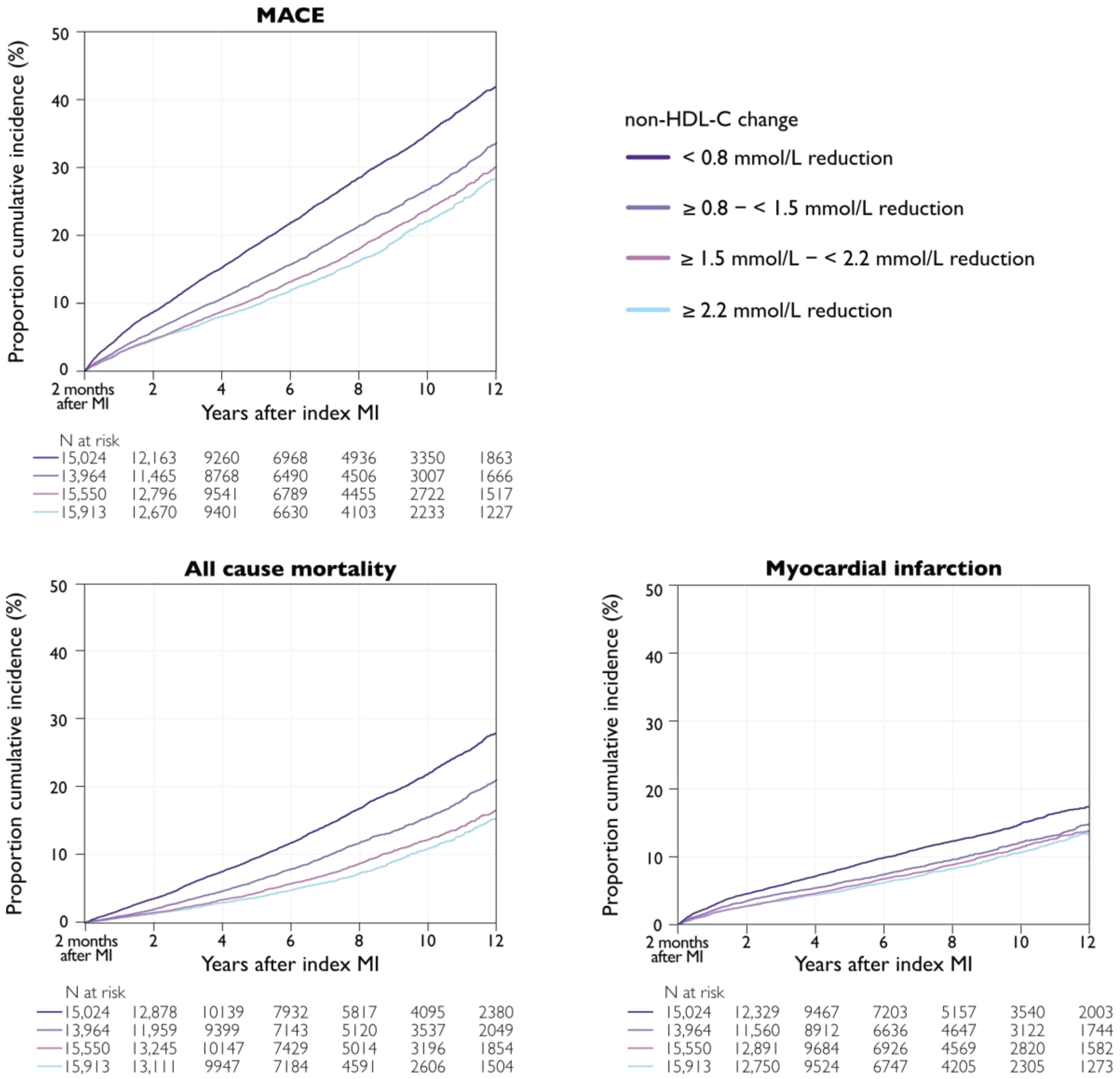

Cumulative incidence rates by outcome and change in non-HDL-C at 2 months after MI. Kaplan–Meier curves of the cumulative incidence rates by quartile non-HDL-C reduction from index MI to follow-up. MACE is the composite outcome of all-cause mortality, MI, or ischaemic stroke. MACE, major adverse cardiovascular event; MI, myocardial infarction; non-HDL-C, non-high-density lipoprotein cholesterol.

Figure S10. Association between quartile change in non-HDL-C between index MI and 2 months and subsequent events

	No. of patients	Event rate (95% CI)	Hazard Ratio (95% CI)	P for trend
**MACE**				
< 0.8 mmol/L reduction	15,024	4.4 (4.2–4.5)	1.00	< 0.001
≥ 0.8 – < 1.5 mmol/L reduction	13,964	3.1 (3.0–3.3)	0.85 (0.80–0.90)	
≥ 1.5 mmol/L – < 2.2 mmol/L reduction	15,550	2.6 (2.5–2.7)	0.74 (0.69–0.78)	
≥ 2.2 mmol/L reduction	15,913	2.4 (2.3–2.5)	0.66 (0.61–0.71)	
**All-cause mortality**				
< 0.8 mmol/L reduction	15,024	2.4 (2.3–2.5)	1.00	< 0.001
≥ 0.8 – < 1.5 mmol/L reduction	13,964	1.6 (1.6–1.7)	0.89 (0.83–0.96)	
≥ 1.5 mmol/L – < 2.2 mmol/L reduction	15,550	1.2 (1.1–1.3)	0.74 (0.68–0.81)	
≥ 2.2 mmol/L reduction	15,913	1.0 (1.0–1.1)	0.68 (0.61–0.76)	
**Myocardial infarction**				
< 0.8 mmol/L reduction	15,024	1.7 (1.6–1.8)	1.00	< 0.001
≥ 0.8 – < 1.5 mmol/L reduction	13,964	1.3 (1.3–1.4)	0.86 (0.79–0.94)	
≥ 1.5 mmol/L – < 2.2 mmol/L reduction	15,550	1.2 (1.1–1.3)	0.75 (0.68–0.82)	
≥ 2.2 mmol/L reduction	15,913	1.1 (1.1–1.2)	0.63 (0.56–0.70)	

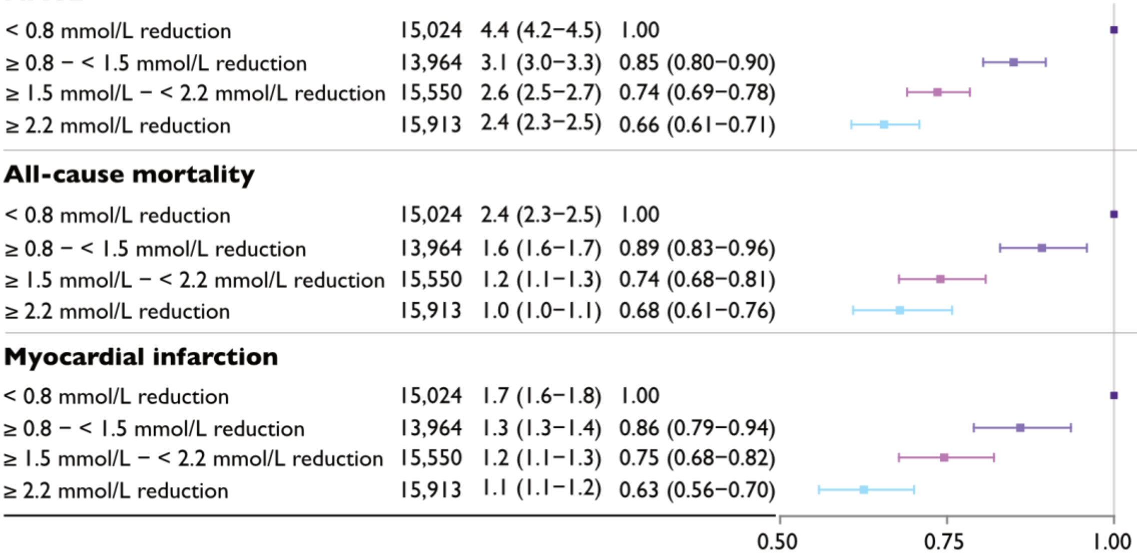

Event rates per 100 person years and hazard ratios by non-HDL-C quartile change between index MI and 2-month follow-up, adjusted for age at follow-up, statin intensity at admission, systolic blood pressure at follow-up, smoking at follow-up, sex, statin intensity at follow-up, body mass index at follow-up, history of diabetes, creatinine at admission, non-HDL-C at admission, and left ventricular ejection fraction at admission. MACE is the composite outcome of all-cause mortality, MI, or ischaemic stroke. MACE, major adverse cardiovascular event; MI, myocardial infarction; non-HDL-C, non-high-density lipoprotein cholesterol.

Figure S11. Achieved non-HDL-C at 2 months and subsequent events

	No. of patients	Event rate (95% CI)	Hazard Ratio (95% CI)	P for trend
**MACE**				
> 2.9 mmol/L	14,394	3.6 (3.4–3.7)	1.00	< 0.001
> 2.4 – ≤ 2.9 mmol/L	12,969	3.0 (2.9–3.2)	0.85 (0.81–0.90)	
> 1.9 – ≤ 2.4 mmol/L	17,213	3.0 (2.9–3.1)	0.82 (0.78–0.86)	
≤ 1.9 mmol/L	15,876	2.8 (2.7–3.0)	0.74 (0.69–0.78)	
**All-cause mortality**				
> 2.9 mmol/L	14,394	1.8 (1.7–1.8)	1.00	< 0.001
> 2.4 – ≤ 2.9 mmol/L	12,969	1.5 (1.4–1.6)	0.84 (0.78–0.90)	
> 1.9 – ≤ 2.4 mmol/L	17,213	1.5 (1.5–1.6)	0.84 (0.78–0.90)	
≤ 1.9 mmol/L	15,876	1.5 (1.4–1.6)	0.78 (0.72–0.85)	
**Myocardial infarction**				
> 2.9 mmol/L	14,394	1.6 (1.5–1.7)	1.00	< 0.001
> 2.4 – ≤ 2.9 mmol/L	12,969	1.3 (1.3–1.4)	0.85 (0.79–0.92)	
> 1.9 – ≤ 2.4 mmol/L	17,213	1.3 (1.2–1.3)	0.80 (0.74–0.86)	
≤ 1.9 mmol/L	15,876	1.1 (1.1–1.2)	0.70 (0.64–0.77)	

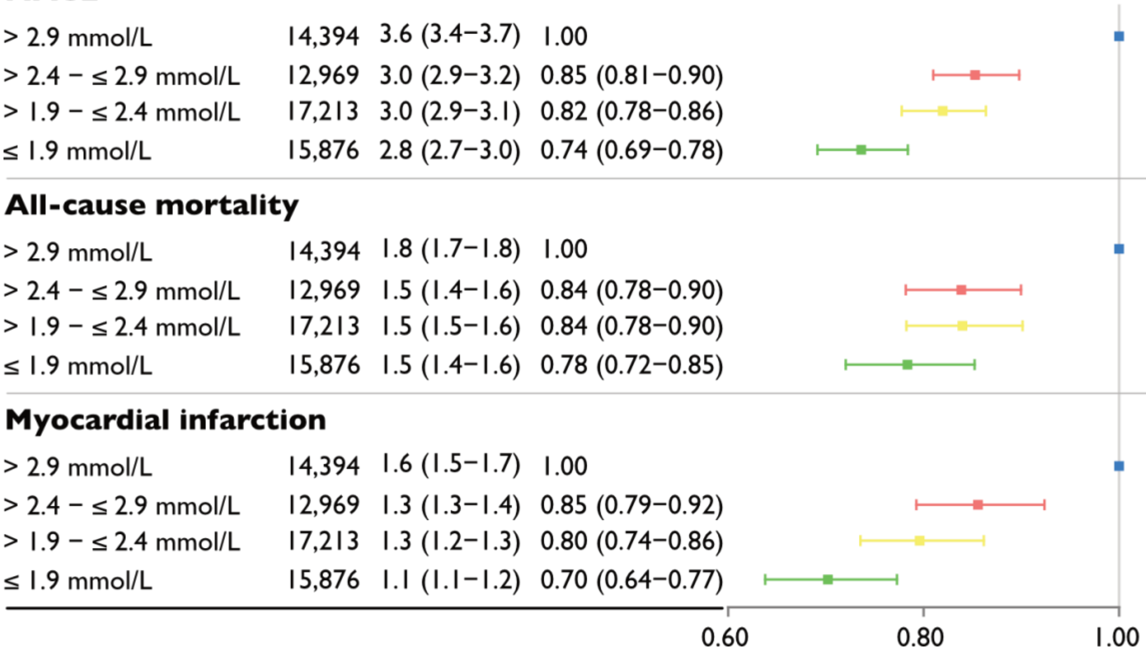

Event rates per 100 person years and hazard ratios by quartiles of achieved non-HDL-C between index MI and at 2-month follow-up, adjusted for age at follow-up, statin intensity at admission, systolic blood pressure at follow-up, smoking at follow-up, sex, statin intensity at follow-up, body mass index at 2-month follow-up, history of diabetes, creatinine at admission, non-HDL-C at admission, and left ventricular ejection fraction at admission. MACE is the composite outcome of all-cause mortality, MI, or ischaemic stroke. MACE, major adverse cardiovascular event; MI, myocardial infarction; non-HDL-C, non-high-density lipoprotein cholesterol.

Every 1 mmol/L reduction in non-HDL-C at 1 year was associated with a 14% lower risk for MACE (HR 0.86, 95% CI 0.84–0.88), 9% lower risk for all-cause mortality (HR 0.91, 95% CI 0.88–0.94), and 18% lower risk for MI (HR 0.82, 95% CI 0.80–0.85) (*Figure S12A*).

-Patients with a larger reduction in non-HDL-C (75th percentile, 2.2 mmol/L reduction) had a lower risk of all outcomes compared to patients with a smaller reduction (25th percentile, 0.7 mmol/L reduction) (*Figure S12B*). HRs were 0.71 (95% CI 0.66–0.77) for MACE, 0.81 (95% CI 0.73–0.89) for all-cause mortality, and 0.66 (95% CI 0.59–0.73) for MI.

Figure S12. Reduction in non-HDL-C and risk of outcomes

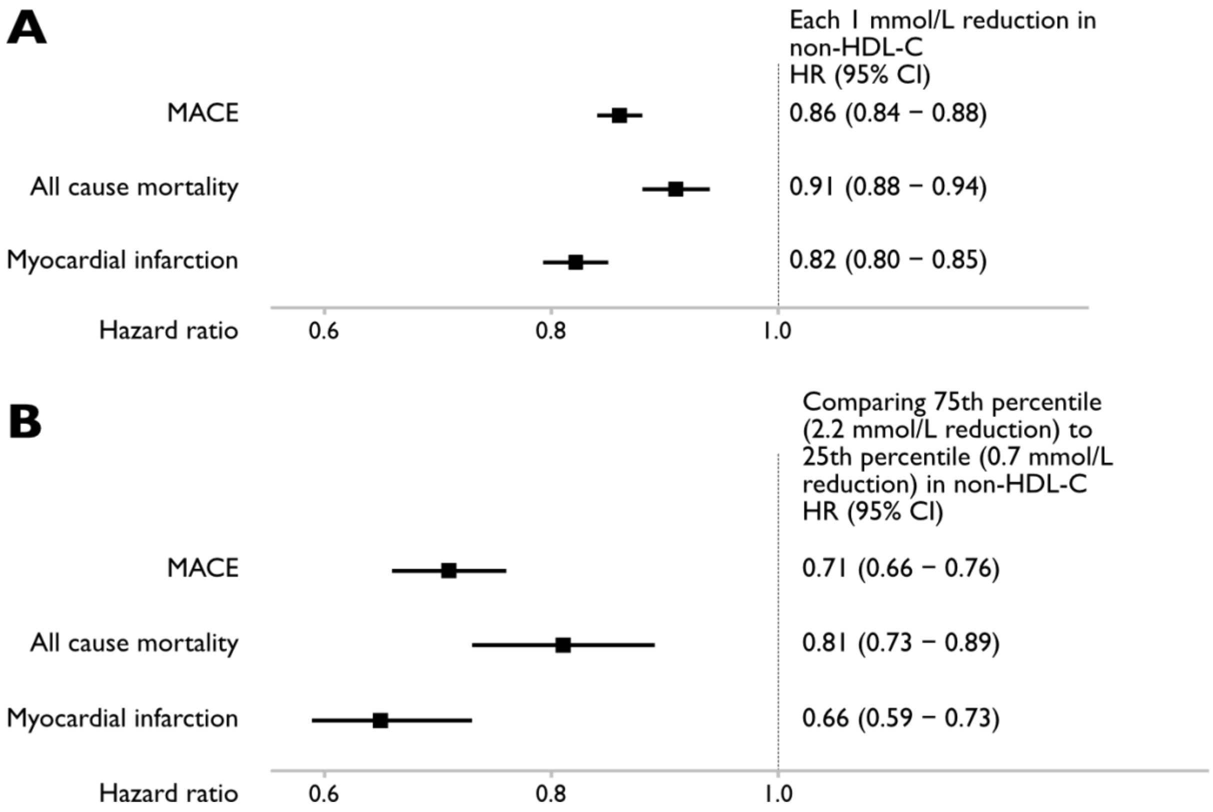

Comparing each 1 mmol/L reduction in non-HDL-C (A) and the 75th percentile of reduction in non-HDL-C to the 25th percentile (B) and hazard ratio (HR) with 95% CI between index MI and 1 year follow-up, adjusted for age at 1-year follow-up, statin intensity at admission, systolic blood pressure at 1-year follow-up, smoking at 1-year follow-up, sex, statin intensity at 1-year follow-up, body mass index at 1-year follow-up, history of diabetes, creatinine at admission, non-HDL-C at admission, and left ventricular ejection fraction at admission. MACE is the composite outcome of all-cause mortality, MI, or ischaemic stroke. CI, confidence interval; HR, hazard ratio; MACE, major adverse cardiovascular event; MI, myocardial infarction; non-HDL-C, non-high-density lipoprotein cholesterol.

Figure S13. Reduction in non-HDL-C at 1-year, complete data

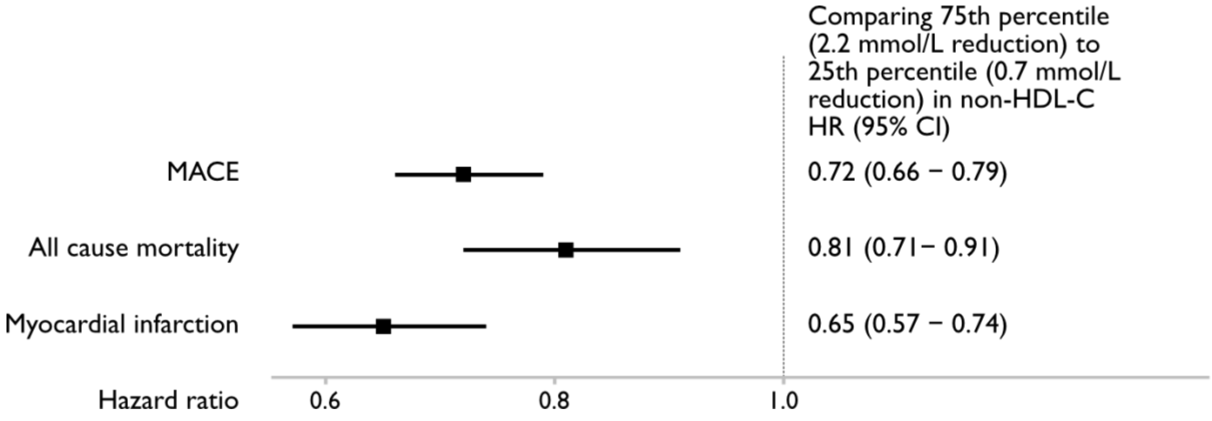

Associations between the 75th percentile of reduction in non-HDL-C and the 25th percentile in patients with complete data: HRs with 95% CIs between index MI and 1 year follow-up, adjusted for age at 1-year follow-up, statin intensity at admission, systolic blood pressure at 1-year follow-up, smoking at 1-year follow-up, sex, statin intensity at 1-year follow-up, body mass index at 1-year follow-up, history of diabetes, creatinine at admission, non-HDL-C at admission and left ventricular ejection fraction at admission. MACE is the composite outcome of all-cause mortality, MI, or ischaemic stroke. CI, confidence interval; HR, hazard ratio; MACE, major adverse cardiovascular event; MI, myocardial infarction; non-HDL-C, non-high-density lipoprotein cholesterol.

Figure S14. 1 mmol/L reduction in non-HDL-C between index MI and 1 year in subgroups

A

MACE	No. of patients	No. of events	Hazard Ratio (95% CI)	P for interaction
**All**				
	56,262	9549	0.86 (0.84–0.88)	
**Diabetes**				0.112
No	47,215	7343	0.85 (0.83–0.88)	
Yes	9047	2206	0.88 (0.85–0.91)	
**BMI (kg/m²)**				0.664
≤ 30	30,621	5254	0.87 (0.84–0.90)	
> 30	10,648	1985	0.86 (0.83–0.90)	
**Sex**				0.211
Men	41,774	6915	0.87 (0.84–0.89)	
Women	14,488	2634	0.85 (0.82–0.88)	
**Statin-use at admission**				0.009
No	48,333	7845	0.85 (0.83–0.87)	
Yes	7929	1704	0.91 (0.87–0.95)	
**eGFR (mL/min/1.73/m²)**				0.892
< 60	3648	1078	0.86 (0.82–0.90)	
≥ 60	52,083	8355	0.86 (0.84–0.88)	
**Age (years)**				0.170
≤ 55	11,091	1214	0.87 (0.83–0.91)	
56–64	16,437	2358	0.88 (0.85–0.91)	
65–70	14,221	2727	0.84 (0.81–0.87)	
71–80	14,331	3174	0.87 (0.84–0.90)	
> 80	182	76	0.93 (0.76–1.12)	

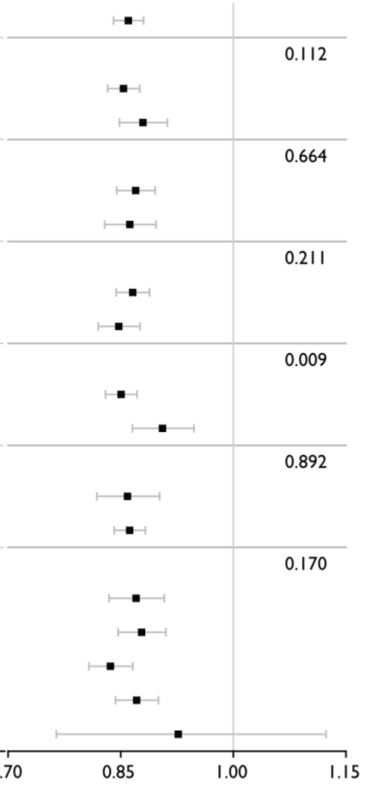

B

All-cause mortality	No. of patients	No. of events	Hazard Ratio (95% CI)	P for interaction
**All**				
	56,262	5427	0.91 (0.88–0.94)	
**Diabetes**				0.029
No	47,215	4054	0.90 (0.87–0.93)	
Yes	9047	1373	0.95 (0.90–1.00)	
**BMI (kg/m²)**				0.753
≤ 30	30,621	2978	0.93 (0.89–0.97)	
> 30	10,648	1070	0.92 (0.87–0.97)	
**Sex**				0.348
Men	41,774	3828	0.92 (0.89–0.95)	
Women	14,488	1599	0.90 (0.86–0.94)	
**Statin-use at admission**				0.002
No	48,333	4387	0.90 (0.87–0.93)	
Yes	7929	1040	0.99 (0.93–1.06)	
**eGFR (mL/min/1.73/m²)**				0.738
< 60	3648	784	0.91 (0.85–0.96)	
≥ 60	52,083	4570	0.92 (0.88–0.95)	
**Age (years)**				0.423
≤ 55	11,091	330	0.93 (0.86–1.01)	
56–64	16,437	1133	0.91 (0.86–0.96)	
65–70	14,221	1682	0.89 (0.85–0.94)	
71–80	14,331	2221	0.94 (0.90–0.98)	
> 80	182	61	0.95 (0.76–1.18)	

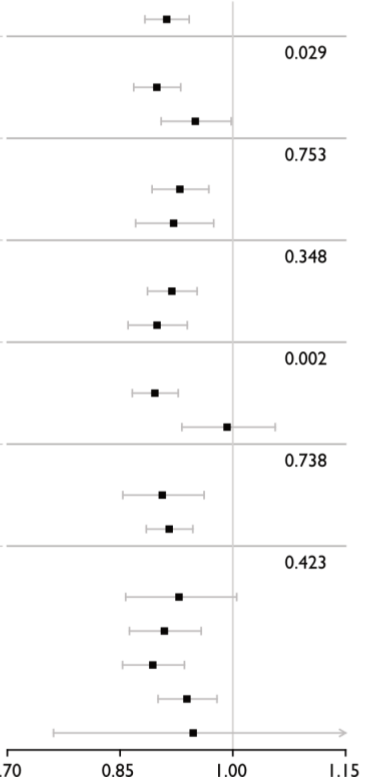

Figure S14 continued. 1 mmol/L reduction in non-HDL-C between index MI and 1 year in subgroups

C

Myocardial infarction	No. of patients	No. of events	Hazard Ratio (95% CI)	P for interaction
**All**				
	56,262	3946	0.82 (0.80–0.85)	
**Diabetes**				0.937
No	47,215	3062	0.82 (0.79–0.85)	
Yes	9047	884	0.82 (0.78–0.87)	
**BMI (kg/m²)**				0.878
≤ 30	30,621	2159	0.82 (0.79–0.86)	
> 30	10,648	887	0.83 (0.78–0.88)	
**Sex**				0.175
Men	41,774	2909	0.83 (0.80–0.86)	
Women	14,488	1037	0.80 (0.76–0.84)	
**Statin-use at admission**				0.291
No	48,333	3270	0.82 (0.79–0.85)	
Yes	7929	676	0.85 (0.79–0.91)	
**eGFR (mL/min/1.73/m²)**				0.420
< 60	3648	361	0.85 (0.78–0.92)	
≥ 60	52,083	3546	0.82 (0.79–0.85)	
**Age (years)**				0.324
≤ 55	11,091	824	0.83 (0.79–0.88)	
56–64	16,437	1123	0.85 (0.81–0.90)	
65–70	14,221	1011	0.80 (0.75–0.84)	
71–80	14,331	969	0.81 (0.76–0.85)	
> 80	182	19	0.87 (0.60–1.28)	

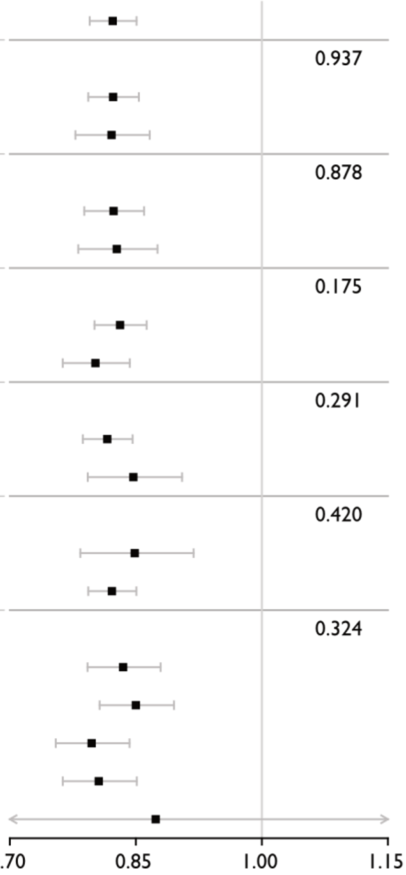

Association between MACE (A) all-cause mortality (B) and non-fatal MI (C) and 1 mmol/L reduction in non-HDL-C at 1 year after index MI, adjusted for age at 1-year follow-up, statin intensity at admission, systolic blood pressure at 1-year follow-up, smoking at 1-year follow-up, sex, statin intensity at 1-year follow-up, body mass index at 1-year follow-up, history of diabetes, creatinine at admission, non-HDL-C at admission, and left ventricular ejection fraction at admission. eGFR, estimated glomerular filtration rate calculated by the Chronic Kidney Disease Epidemiology Collaboration equation. MACE is the composite outcome of all-cause mortality, MI, or ischaemic stroke. MACE, major adverse cardiovascular event; MI, myocardial infarction; non-HDL-C, non-high-density lipoprotein cholesterol.

Figure S15. Association between achieved non-HDL-C levels at 1 year and outcomes in different adjustment models

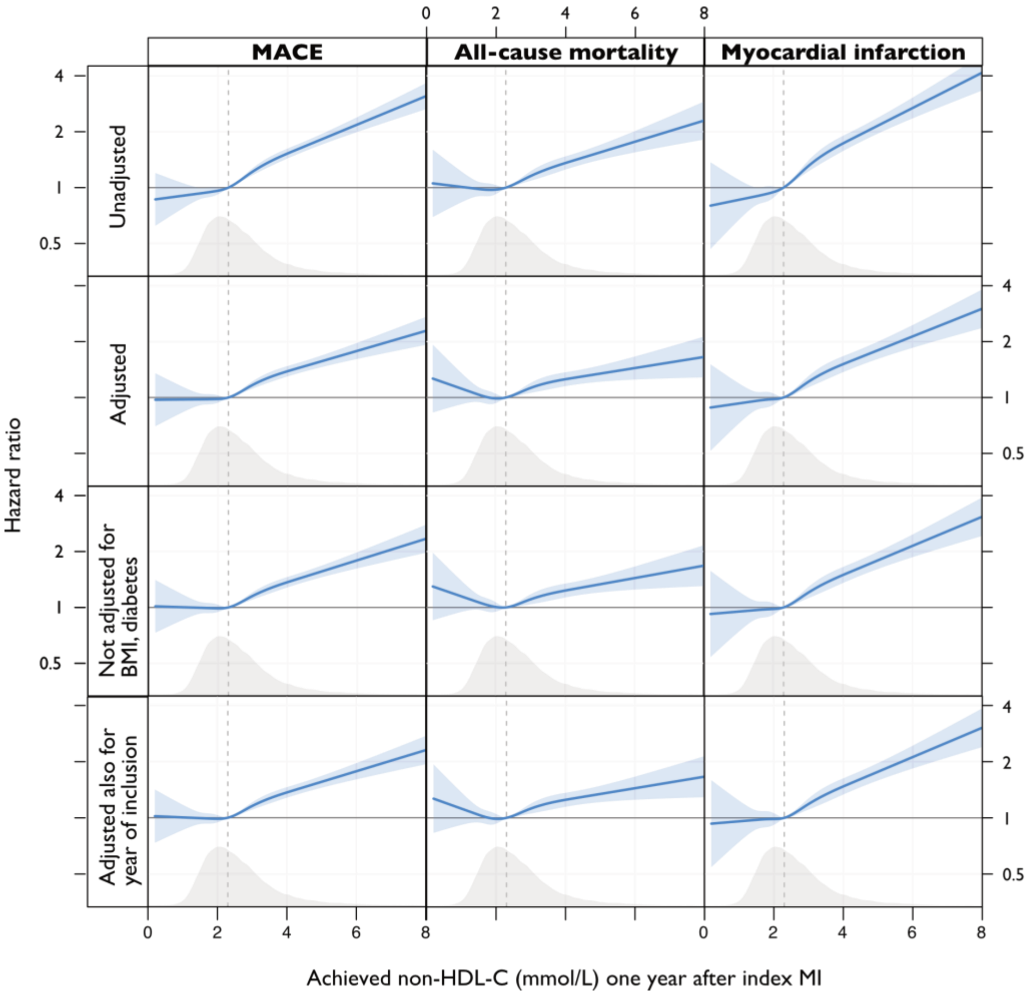

Association between achieved values of non-HDL-C and outcomes. Models are adjusted for age at 1-year follow-up, systolic blood pressure at 1-year follow-up, smoking at 1-year follow-up, sex, non-HDL-C at admission, left ventricular ejection fraction at admission, creatinine at admission, statin intensity at admission, statin intensity at 1-year follow-up, body mass index at 1-year follow-up, and history of diabetes. HRs with 95% CIs. Vertical dashed lines indicate the reference level (i.e., HR of 1 at median non-HDL-C 2.3 mmol/L). The grey area shows the population distribution (density) of achieved non-HDL-C. MACE is the composite outcome of all-cause mortality, MI, or ischaemic stroke. CI, confidence interval; HR, hazard ratio; MACE, major adverse cardiovascular event; MI, myocardial infarction; non-HDL-C, non-high-density lipoprotein cholesterol.

Figure S16. Early and late goal achievement by inclusion year stratum

MACE	No. of patients	Event rate (95% CI)	Hazard Ratio (95% CI)	P for trend
**Study inclusion 2005–2014**				
≥ 2.2 mmol/L	13,962	3.4 (3.3–3.5)	1.00	<0.001
< 2.2 mmol/L at 2 months	2317	3.2 (2.9–3.4)	0.88 (0.81–0.97)	
< 2.2 mmol/L at 1 year	1751	3.0 (2.8–3.3)	0.88 (0.79–0.98)	
< 2.2 mmol/L at 2 months and sustained at 1 year	2851	3.0 (2.8–3.2)	0.84 (0.77–0.92)	
**Study inclusion 2015–2021**				
≥ 2.2 mmol/L	8043	2.6 (2.5–2.8)	1.00	<0.001
< 2.2 mmol/L at 2 months	3062	2.5 (2.1–2.8)	0.84 (0.72–0.98)	
< 2.2 mmol/L at 1 year	5299	2.3 (2.0–2.5)	0.87 (0.76–0.99)	
< 2.2 mmol/L at 2 months and sustained at 1 year	9233	2.3 (2.1–2.5)	0.76 (0.67–0.86)	

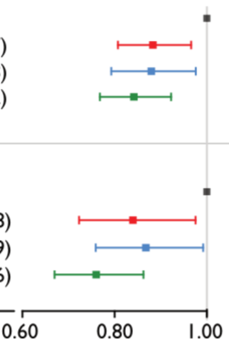

Adjusted cox proportional hazard models and event rates for MACE per 100 person years for early and late goal achievement divided by study inclusion before 2005-2014, and 2015-2021, respectively. Adjusted for age at 1-year follow-up, statin intensity at admission, systolic blood pressure at 1-year follow-up, smoking at 1-year follow-up, sex, statin intensity at 1-year follow-up, body mass index at 1-year follow-up, history of diabetes, creatinine at admission, non-HDL-C at admission, and left ventricular ejection fraction at admission. MACE is the composite outcome of all-cause mortality, MI, or ischaemic stroke. MACE, major adverse cardiovascular event.

Disclosures of interest

J.S.: institutional grant from Pfizer during the conduct of the study. B.L., H.M., J.W., no conflict of interest to disclose. E.H.: institutional grants from Amgen, Pfizer, and honoraria from Amgen, NovoNordisk, Amarin, Sanofi, NovoNordisk, Novartis, Bayer, and Astra Zeneca outside the submitted work. A.M. and N.C. are employed by Pfizer. J.B.: institutional grants from Amgen, Novartis, Akcea Therapeutics outside the submitted work. M.L: Institutional grants and honoraria from Astra Zeneca, Sanofi, Amarin and Amgen outside the submitted work. KKR Institutional grants Amgen, Sanofi, Daiichi Sankyo, Ultragenix, Amarin, Consultancy: Amgen, Sanofi, Regeneron, Pfizer, Viatris, Abbott, AstraZeneca, Lilly, Kowa Pharmaceutics, Novo Nordisk, Boehringer Ingelheim, Esperion, Cargene Therapeutics, Resverlogix, Novartis, Silence Therapeutics, NewAmsterdam Pharma, Scribe Therapeutics, CRISPR Therapeutics, VAXXINITY, Amarin, CSL Behring, Bayer, Cleerly Health, Emendobio and Stock Options PEMI31, SCRIBE, New Amsterdam Pharma.

Author contributions

All listed authors were involved in study conceptualization and/or interpretation of the study findings. Additionally, each author contributed to the drafting of the work or critical revision for important intellectual content. All authors approve the final version for publication and agree to be accountable for all aspects of the work.

J.S. Conceptualization: Equal
Investigation: Equal
Methodology: Equal
Project administration: Equal
Validation: Equal
Visualization: Equal
Writing – original draft: Lead
Writing – review & editing: Equal

M.L. Investigation: Equal
Writing – review & editing: Equal

K.R. Investigation: Equal
Writing – review & editing: Equal
Writing – review & editing: Equal

B.L. Investigation: Equal
Supervision: Supporting
Writing – review & editing: Equal

J.W. Data curation: Lead
Formal analysis: Lead
Methodology: Equal
Software: Lead
Visualization: Equal

H.M. Investigation: Equal
Supervision: Supporting

A.M. Investigation: Equal
Writing – review & editing: Equal

N.C. Investigation: Equal
Writing – review & editing: Equal

J.B. Investigation: Equal
Writing – review & editing: Equal

E.H. Conceptualization: Equal
Funding acquisition: Lead
Investigation: Equal
Methodology: Equal
Project administration: Equal
Resources: Lead
Supervision: Lead
Validation: Equal
Visualization: Equal
Writing – original draft: Equal
Writing – review & editing: Equal

Supplementary References

1. Martin SS, Blaha MJ, Elshazly MB, Toth PP, Kwiterovich PO, Blumenthal RS, et al. Comparison of a novel method vs the Friedewald equation for estimating low-density lipoprotein cholesterol levels from the standard lipid profile. *JAMA* 2013;310:2061–2068. doi: https://doi.org/10.1001/jama.2013.280532

2. Schubert J, Lindahl B, Melhus H, Renlund H, Leosdottir M, Yari A, et al. Low-density lipoprotein cholesterol reduction and statin intensity in myocardial infarction patients and major adverse outcomes: a Swedish nationwide cohort study. *Eur Heart J* 2021;42:243–252. doi: https://doi.org/10.1093/eurheartj/ehaa1011

3. Byrne RA, Rossello X, Coughlan JJ, Barbato E, Berry C, Chieffo A, et al. ESC Scientific Document Group. 2023 ESC Guidelines for the management of acute coronary syndromes. *Eur Heart J Acute Cardiovasc Care* 2023;13:55–161. doi: https://doi.org/10.1093/ehjacc/zuad107

4. Sniderman AD. Differential response of cholesterol and particle measures of atherogenic lipoproteins to LDL-lowering therapy: implications for clinical practice. *J Clin Lipidol* 2008;2:36–42. doi: https://doi.org/10.1016/j.jacl.2007.12.006

5. Thanassoulis G, Williams K, Ye K, Brook R, Couture P, Lawler PR, et al. Relations of Change in Plasma Levels of LDL-C, Non-HDL-C and apoB With Risk Reduction From Statin Therapy: A Meta-Analysis of Randomized Trials. *J Am Heart Assoc* 2014;3:e000759. doi: https://doi.org/10.1161/jaha.113.000759

6. Karlson BW, Palmer MK, Nicholls SJ, Lundman P, Barter PJ. Doses of rosuvastatin, atorvastatin and simvastatin that induce equal reductions in LDL-C and non-HDL-C: Results from the VOYAGER meta-analysis. *Eur J Prev Cardiolog* 2016;23:744–747. doi: https://doi.org/10.1177/2047487315598710

7. Schubert J, Lindahl B, Melhus H, Renlund H, Leosdottir M, Yari A, et al. Elevated low-density lipoprotein cholesterol: An inverse marker of morbidity and mortality in patients with myocardial infarction. *J Intern Med* 2023;294:616–627. doi: https://doi.org/10.1111/joim.13656

8. Johannesen CDL, Mortensen MB, Langsted A, Nordestgaard BG. Apolipoprotein B and Non-HDL Cholesterol Better Reflect Residual Risk Than LDL Cholesterol in Statin-Treated Patients. *J Am Coll Cardiol* 2021;77:1439–1450. doi: https://doi.org/10.1016/j.jacc.2021.01.027
